# Genome of an allotetraploid wild peanut *Arachis monticola*: a de novo assembly

**DOI:** 10.1093/gigascience/giy066

**Published:** 2018-06-19

**Authors:** Dongmei Yin, Changmian Ji, Xingli Ma, Hang Li, Wanke Zhang, Song Li, Fuyan Liu, Kunkun Zhao, Fapeng Li, Ke Li, Longlong Ning, Jialin He, Yuejun Wang, Fei Zhao, Yilin Xie, Hongkun Zheng, Xingguo Zhang, Yijing Zhang, Jinsong Zhang

**Affiliations:** 1 College of Agronomy, Henan Agricultural University, Zhengzhou 450002, China; 2 Biomarker Technologies Corporation, Beijing 101300, China; 3 State Key Lab of Plant Genomics, Institute of Genetics and Developmental Biology, Chinese Academy of Sciences, Beijing 100101, China; 4 National Key Laboratory of Plant Molecular Genetics, CAS Center for Excellence in Molecular Plant Sciences, Institute of Plant Physiology and Ecology, Shanghai Institutes for Biological Sciences, Chinese Academy of Sciences, Shanghai 200032, China

## Abstract

*Arachis monticola* (2n = 4x = 40) is the only allotetraploid wild peanut within the *Arachis* genus and section, with an AABB-type genome of ∼2.7 Gb in size. The AA-type subgenome is derived from diploid wild peanut *Arachis duranensis*, and the BB-type subgenome is derived from diploid wild peanut *Arachis ipaensis*. *A. monticola* is regarded either as the direct progenitor of the cultivated peanut or as an introgressive derivative between the cultivated peanut and wild species. The large polyploidy genome structure and enormous nearly identical regions of the genome make the assembly of chromosomal pseudomolecules very challenging. Here we report the first reference quality assembly of the *A. monticola* genome, using a series of advanced technologies. The final whole genome of *A. monticola* is ∼2.62 Gb and has a contig N50 and scaffold N50 of 106.66 Kb and 124.92 Mb, respectively. The vast majority (91.83%) of the assembled sequence was anchored onto the 20 pseudo-chromosomes, and 96.07% of assemblies were accurately separated into AA- and BB- subgenomes. We demonstrated efficiency of the current state of the strategy for de novo assembly of the highly complex allotetraploid species, wild peanut (*A. monticola*), based on whole-genome shotgun sequencing, single molecule real-time sequencing, high-throughput chromosome conformation capture technology, and BioNano optical genome maps. These combined technologies produced reference-quality genome of the allotetraploid wild peanut, which is valuable for understanding the peanut domestication and evolution within the *Arachis* genus and among legume crops.

## Introduction

Peanut (*Arachis hypogaea* L.) is widely cultivated in subtropical and tropical regions as a plant-based resource for protein and edible oil, which has a key role in global food security. The genus *Arachis* is unique for its subterranean fruit, which originated in South America and has ∼80 described species divided into nine sections based on their morphology, cross compatibility relationships, and geographical distribution [1]. Section *Arachis* is of particular interest because it contains 30 diploid wild species, one tetraploid wild species (*A. monticola*), and cultivated peanut (*A. hypogaea*) (2n = 4x = 40). *A. monticola* was distinct from accessions of *A. hypogaea* with high genetic identity [2, 3]. Moreover, hybrids between *A. monticola* and *A. hypogaea* are fertile [4]. *A. monticola* is considered a distinct species from *A. hypogaea* based mainly on its fruit structure, which has an isthmus separating each seed, resembling the diploid wild species [5, 6]. Comparison of the genomes among the *A. monticola*, *A. hypogaea*, and wild species should shed light on the evolutionary and/or domesticated events in the cultivated species are undergoing.

As a relatively young allotetraploid species, the genome of wild peanut *A. monticola* exhibits complexity with an AABB-type genome of ∼2.7 Gb [7] and shares many regions of high similarity with its two subgenomes [8]. Challenges are present for its genome assembly due to the large polyploid genome structure and highly homologous genomic sequences. Because of these difficulties, sequencing of the diploid ancestors *A. ipaensis* and *A. duranensis* was first completed [8]. The total assembled genome sizes were 1.025 Gb and 1.338 Gb, respectively, for the two species, with a N50 contig length of 22 Kb, using paired-end Illumina sequencing. All *A. ipaensis* pseudomolecules were larger than their *A. duranensis* counterparts, and *A. ipaensis* may be a direct descendant contributing to the B subgenome of the cultivated peanut [8]. Although previous publications of reference genome sequences of peanut diploid ancestors (*A. ipaensis* and *A. duranensis*) provide valuable insight and knowledge of peanut/legumes and have facilitated peanut research, all the cultivated peanut varieties are allotetraploids. A high-quality reference genome of an allotetraploid peanut is important for evolution, origin, and domestication research of wild and cultivated peanuts and a favorable resource for peanut breeding, making it an important target for the entire peanut research community.

In this study, we used a series of advanced technologies, including whole-genome shotgun sequencing, single molecule real-time (SMRT) sequencing, high-throughput chromosome conformation capture (Hi-C) technology, and BioNano optical genome mapping, to generate a high-quality genome sequence for the tetroploid wild peanut species *A. monticola*. By combining these very long reads with highly accurate short reads, we have been able to produce an assembly of this tetroploid wild species (*A. monticola*) genome. In total, we used 767.25 billion bases and 210.83-fold genome coverage of BioNano data for the genome assembly. Finally, we generated a 2.62-Gb assembly, spanning 97% of the estimated genome size for *A. monticola*.

## Results

**Figure 1:**
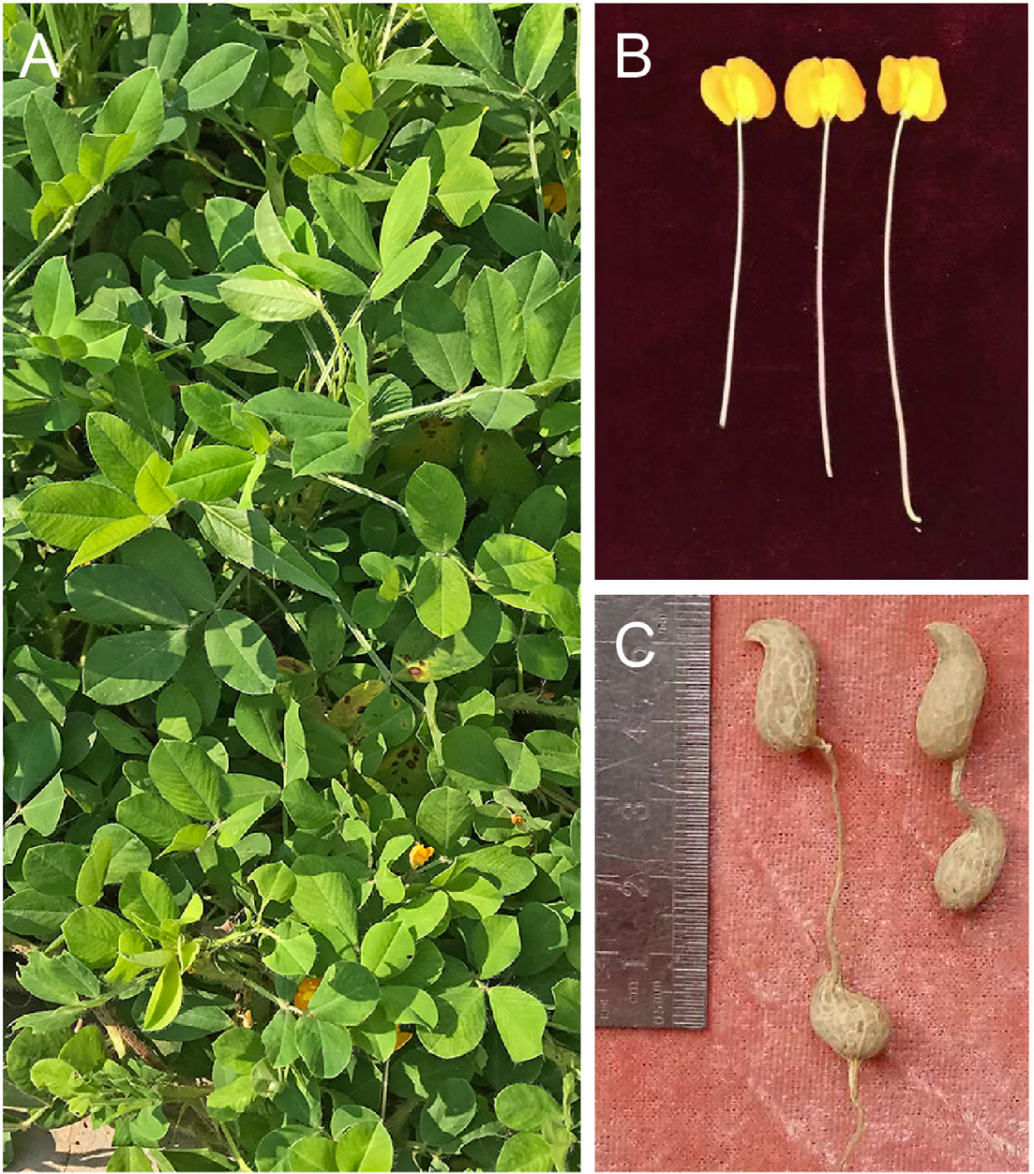
Morphological characters of the *Arachis monticola*. Mature plants in field (**A**), flowers (**B**), and pods (**C**) are shown.

*A. monticola* is an allotetraploid wild peanut species and has features different from the tetraploid cultivated peanut (Fig. 1). Line PI 263393 was selected for genome sequencing. The peanut plants were grown in a growth chamber at 25°C, and DNA was extracted from fresh leaves of 30-day-old wild peanut seedlings. To create the *A. monticola* genome assembly, we generated four extremely large primary data sets including 462.87 Gb Illumina reads (Supplemetary Table S1a), 11.5 million SMRT long reads as ∼91.71 Gb (Supplemetary Table S1b), 2.88 million (∼596.26 Gb) high-quality BioNano optical molecules (Supplemetary Table S1c), and 76.54-fold coverage of the genome of Hi-C data (Supplemetary Table S1d). All the reads were generated from the same *A. monticola* line. Taking advantage of integrated technologies, we achieved 2.62 Gb high-quality reference genome of wild peanut with 20 pseudo-chromosomes (Table 1 and Supplemetary Table S2c) and successfully distinguished two subgenomes (*A.mon-A* and *A.mon-B*) corresponding to its diploid progenitors, *A. ipaensis* and *A. duranensis*, respectively (Supplemetary Table S2d).

**Table 1:** Statistics of pseudochromosomes of *A. monticola*

	Chr	Length (bp)	No. of gap	Gap length (bp)	Gaps ratio (%)	Anchored percent (%)
*A.mon*-A	A.mon-A01	118,283,061	1961	12,923,146	10.93	4.51
	A.mon-A02	84,409,872	1598	13,652,890	16.17	3.22
	A.mon-A03	123,011,103	2089	18,448,429	15.00	4.69
	A.mon-A04	106,244,467	2020	15,031,534	14.15	4.05
	A.mon-A05	123,320,146	1950	15,552,662	12.61	4.70
	A.mon-A06	98,474,784	1770	11,764,791	11.95	3.75
	A.mon-A07	72,108,480	1299	7250,302	10.05	2.75
	A.mon-A08	39,681,652	442	1898,702	4.78	1.51
	A.mon-A09	107,717,523	1889	11,324,084	10.51	4.11
	A.mon-A10	100,634,791	1847	13,895,555	13.81	3.84
	Un-chr	61,870,352	422	7811,614	12.63	2.36
*A.mon*-B	A.mon-B01	140,073,190	2773	17,354,378	12.39	5.34
	A.mon-B02	124,915,013	2271	14,941,271	11.96	4.76
	A.mon-B03	160,549,902	2512	18,727,668	11.66	6.12
	A.mon-B04	147,957,427	2521	16,939,677	11.45	5.64
	A.mon-B05	121,568,645	2396	14,347,666	11.80	4.63
	A.mon-B06	154,488,041	2644	22,222,939	14.38	5.89
	A.mon-B07	136,067,974	2462	15,804,193	11.61	5.19
	A.mon-B08	138,850,997	2492	17,429,178	12.55	5.29
	A.mon-B09	163,848,611	2991	16,573,361	10.12	6.24
	A.mon-B10	147,468,805	2693	18,369,757	12.46	5.62
	Un-chr	49,370,401	428	7142,698	14.47	1.88
Unknown	–	103,005,886	972	16,282,706	15.81	–
	Total	2623,921,123	46,879	325,689,201	12.41	–

### Initial genome assembly

An independent whole-genome sequencing assembly was executed using Allpath-LG v1.4 (Allpath-LG, RRID:SCR_010742) [9] to increase the lengths of scaffolds and to fill gaps in the *A. monticola* assembly. Eleven paired-end and mate-paired libraries ranging from 200 bp to 17 kb were constructed and sequenced (Supplementary Table S1a). From 171-fold coverage reads (∼462.87 Gb), we assembled into 1.66 Gb results with scaffold N50 and contig N50 of 369.06 kb and 16.17 kb, respectively (Supplementary Table S2a).

We also assembled the *A. monticola* genome using 97.71-Gb-long Pacific Biosciences (PacBio) reads, covering approximately 36.10-fold coverage of genome size (Supplementary Table S1b). Because of a high error rate of PacBio reads, we first corrected these by the error correction module of Canu v1.5 [10] based on 36.10 x Pacbio subreads. For subreads aborted by Canu, we corrected them with LoRDEC v0.5 [11] based on ∼50-fold coverage of Illumina short reads. Finally, we retained 34.07-fold coverage of high-quality subreads (92.78 Gb) and independently assembled them with Falcon v0.7 [12], WTDBG v1.2.8 [13], and Canu v1.5 [10]. The assembled sizes from Falcon, WTGDB, and Canu are 1.88 Gb, 1.96 Gb, and 2.26 Gb, respectively. The contig N50 of assembly results was 81.5 kb, 82.8 kb, and 109.2 kb, respectively, for the three methods (Supplementary Table S2b). The completeness assessment of these assembles through Benchmarking Universal Single-Copy Orthologs (BUSCO) databases (BUSCO, RRID:SCR_015008) [14] and Core Eukaryotic Genes Mapping Approach (CEGMA, RRID:SCR_015055) [15] showed that more than 96% core eukaryotic genes (CEGs) and 90% of complete BUSCOs are detectable, suggesting the high completeness of the assembly results. We then polished the consensus sequence of three assemblies based on 50x Illumina pair-end reads using Pilon v1.22 software [16]. To take advantage of assemblies from different tools and generate more contiguity and connectivity results, we merged them together with quickmerge v0.2 package [17]. The strict conditions were considered in this step to avoid chimeric errors. We obtained a genome of 2.24 Gb with contig N50 and longest contig of 120.61 kb and 1.89 Mb, respectively (Supplementary Table S2b).

### Physical map construction

To develop a robust physical map for the allotetraploid wild peanut that could be helpful to place sequence contigs on chromosomes and to determine the physical length of gaps between them [18], we constructed BioNano optical genome map libraries for the sequencing genotype from the fresh leaves. From the enzyme density and distribution assessment of genome sequences using Label Density Calculator v1.3.0 (BioNano Genomics, CA, USA), we adopted the Nt.BspQI nickase for optical map library construction. The basic process of BioNano raw data was conducted using the IrysView v2.5.1 package (BioNano Genomics). Molecules whose lengths are more than 150 kb (with label SNR > = 3.0 and average molecule intensity <0.6) were retained for further genome assembling. We obtained 2.8 million (∼596.26 Gb) high-quality optical molecules, accounting for ∼210x coverage of genome size (Supplementary Table S1c). The N50 of the molecules is 210.83 Kb (Supplementary Table S1c). On the basis of the label positions on single DNA molecules, de novo assembly was performed by a pairwise comparison of all single molecules and overlap-layout-consensus path building, which was adopted by IrysView v2.5.1 assembler [19]. The parameter set for large genomes was used for assembly with the IrysView software. We considered only molecules containing more than seven nicking enzyme sites for assembly (min label per molecule: 8). A *P* value threshold of 1e-8 was used during the pairwise assembly, and 1e-9 for extension and refinement steps and 1e-12 for merging contigs were adopted. The resulting physical map covers approximately 2.65 Gb (around 98.15% of the 2.7-Gb genome size). We generated 1,404 optical map-based scaffolds with N50 of 3.4 Mb for *A. monticola* (Supplementary Table S1c). The high-quality optical map would be used for genome curation and hybrid assembly with SMRT-based assembly, combing the meta-pair (MP) links and Hi-C data.

### Scaffold construction and curation

A total of nine mate-pair libraries ranging from 3-kb to 17-kb fragments were prepared for scaffolds, which accounted for ∼132-fold coverage of the previous estimated genome size (2.7 Gb) [7] (Supplementary Table S1a). To decrease chimeras in the initial assembly results, we mapped the different fragment mate-paired data to the contigs using BWA v0.7.10 (BWA, RRID:SCR_010910) [20], considering only unique mapping reads for further scaffold construction. Further scaffolding was performed by SSPACE v3.0 (SSPACE, RRID:SCR_005056) [21]. Contigs are assembled into scaffolds with MP information, estimating gaps between the contigs according to the distance of MP links. Two contigs supported by at least three reasonable MP links in each fragment library (insert size ± 5 SD) were joined as a scaffold. We assembled 29,454 contigs into 9,157 scaffolds with large reasonable intra-gaps sequences (Supplementary Table S2c). In this step, we obtained 2.35 Gb assembly results for *A. monticola*, whose scaffold N50 and L50 are 491.06 kb and 1,396 kb, respectively (Supplementary Table S2c).

As a relatively young allotetraploid species, the genome of *A. monticola* is particularly complicated, especially considering the phenomenon of partially homologous sequences between its two subgenomes [8, 22]. The assembly results of allotetraploid genome from SMRT reads may introduce lots of chimeric errors from high homologous and/or large repeated regions of *A. monticola*. The optical map of single molecules from BioNano Genomics’ Irys System could assemble large homologous and repeated regions, taking advantage of its super long molecule reads. As a result, detection of conflicts between contigs/scaffolds and genome map, and correction of the potential errors are strongly necessary and feasible.

To ascertain the quality of assembly results, we generated an *in silico* map of merged results using Knickers v1.5.5.0 program [19] with Nt.BspQI nickase. From the comparison between the contigs/scaffolds and optical maps by RefAligner v5122 [19], we identified 610 conflicts. Next Generation Mapping was used to resolve conflicts between the sequence and optical map assemblies by breaking conflict point of assembly. Conflicts were identified based on chimeric score of a conflict junction, mate-pairs information, and SMRT molecules alignment results, which is near the conflict junctions on the optical genome map. The chimeric score of conflict junction is defined as the percentage of BioNano molecules that fully align to the 50-kb flanks of optical map. If the chimeric scores of the conflict junction were ≥30 and more than two fully aligned optical molecules located across the conflict junction of genome map, we suggested a candidate chimerical error in scaffold/contig sequence. The alignment results of conflict regions were visualized in IrysView [19] for manual investigation. Knickers, RefAligner, and IrysView were obtained from BioNano Genomics [19]. Further investigation of mate-paired links and SMRT molecules alignment would assist to make a decision of cutting on selected sequences. If the mate-pair relationship (3 ∼ 17 kb) of 10-kb flanks of conflict junction is in disagreement or <5 coverage of fully aligned Pacbio molecules are across this region, we suggested breaking the point. We considered the consistent soft-clip sites of SMRT molecules on reference sequence as accurate break point. All proposed cuts were manually evaluated using BioNano molecule-to-genome map alignments, SMRT molecule-to-sequence contig alignments, and mate-paired libraries mapping results based on integrated graphic platform. Of these conflicts, 600 were chimeric in the long reads assembly, and 10 were left unresolved. After chimeric correction, we assembled the 6,262 hybrid scaffolds based on genome map hybrid assembly. The genome size of *A. monticola* is 2.62 Gb, with scaffold N50 of 1.51 Mb (Supplementary Table S2c).

### Gap filling and SMRT-error correction

To improve the contiguity of assembly results, we fulfilled the gap filling process combined SMRT sequencing data, Illumina data. PBJelly [23] was used to fill gaps using approximately 34.07-fold coverage of error-corrected SMRT sequencing data from the initial genome assembly step. Then we further filled retaining gaps using 39-fold coverage pair-end data (Supplementary Table S1a), along with *de Bruijn* graph analysis to detect instances where a unique path of reads spanned a gap, implemented with Gapcloser v1.12 of SOAPDenovo packages (GapCloser, RRID:SCR_015026) [24]. During the gap-filling procedure, 42.87-Mb gaps were filled by SMRT long reads and Illumina data.

To ensure base-pairing accuracy of assembly results from SMRT molecules, we further polished the consensus sequence after the construction of the pseudomolecules based on ∼105 Gb Illumina pair-end reads using Pilon [16]. A total of 5,607 kb bases, including single nucleotide polymorphisms and small Indels, were corrected, of which 0.21% were small indels.

### Pseudomolecules construction and subgenome identification

Hi-C technology enables the generation of genome-wide 3D proximity maps and is an efficient and low-cost strategy for sequences cluster, ordered, and orientation for pseudomolecule construction [25]. This technology has been successfully applied in recent complex genome projects, including goat [26], Tartary buckwheat [27], wild emmer [28], and barely [29]. We constructed three Hi-C fragment libraries ranging from 300 to 700 bp and sequenced them using the Illumina X-TEN platform (Illumina, San Diego, CA, USA) for pseudomolecules construction. Mapping of Hi-C reads and assignment to restriction fragments were performed as described elsewhere [25]. Briefly, adapter sequences of raw reads were trimmed with cutadapt v1.0 (cutadapt, RRID:SCR_011841) [30], and low-quality paired-end (PE) reads were removed for clean data. The clean Hi-C reads, accounting for ∼60-fold coverage of the*A. monticola* genome, were mapped to the assembly results with bwa align v0.7.10 (BWA, RRID:SCR_010910) [20] (Supplementary Table S1d). Only uniquely aligned pairs read whose map quality is >20 were considered for further analysis. Duplicate removal, sorting, and quality assessment were performed with HiC-Pro v2.8.1 [31]. The 21.98% of Hi-C data were valid interaction pairs. Raw counts of Hi-C links were aggregated in 50-kb bins and normalized separately for intra- and inter-chromosomal contacts using LACHESIS [25]. We clustered the sequences into an initial 20 groups according to threshold of the contact frequency. For each group, we clustered the sequences in 5 subgroups and independently decided the order and orientation of sequences based on contact probability of each subgroup. The whole order and orientation subgroup was considered as super-bin and recalculated for the interaction matrices for each group. Then LACHESIS [25] was used to assign the order and orientation of each group. Based on 76.54-fold coverage of Hi-C data, the vast majority (91.83%) of the assembled sequence was anchored onto the 20 pseudo-chromosomes by frequency distribution of valid interaction pairs (Table 1).

Benefiting from the published genomes of *A. duranensi*s and *A. ipaensis*, the donors of alloteraploid peanut, we are able to directly identify the corresponding subgenomes based on the whole genome comparison between the assembly results of *A. monticola* and the two wild diploid peanuts. We aligned the assembly results to its ancestral genomes with Mummer v2.23 [32] and successfully distinguished more than 96.07% of sequences into *A.mon-A* and *A.mon*-B subgenomes (Table 1). Finally, the subgenome size of *A.mon*-A and *A.mon*-B is 1,035.76 Mb and 1,485.16 Mb, respectively, which is comparable to that of their ancestors, *A. duranensi*s and *A. ipaensis* (Table 2; Supplementary Table S2d).

**Table 2:** Comparison of assembly results between *A. monticola* and its progenitors

	*A.mon*-A	*A.mon*-B	*A. duranensis*	*A. ipaensis*
Genome size (bp)	1035,756,231	1485,159,006	1068,326,401	1257,035,815
Contig number	18,620	27,431	135,613	123,165
Max length (bp)	1481,449	1683,058	221,145	250,973
Min length (bp)	14,852	10,392	10,007	10,021
Contig N50 (bp)	107,702	110,501	22,900	22,562
Contig N90 (bp)	29,116	29,291	3342	5216
Gap number	18,005	26,847	134,110	122,617
Gap ratio (%)	12.50	12.11	11.95	7.32
GC content (%)	35.79	36.18	35.81	36.85

Note: only sequences whose length is more than 10 kb are considered.

### Genome quality assessment

Completeness of gene-space representation was evaluated based on the plants dataset of the BUSCO database with the BUSCO pipeline v3.0.2 (BUSCO, RRID:SCR_015008) [14]. The results showed that 91.67% of complete gene models could be detected in the*A. monticola* genome (Supplementary Table S3a). Comparison analysis suggested that the gene region completeness of assemblies is slightly better than their corresponding progenitors (Supplementary Table S3a).

CEGMA [15] provides a simple method to rapidly assess genome completeness. It comprises a set of highly conserved, single-copy genes, present in all eukaryotes, including 458 CEGs, 248 of which are highly conserved CEGs. CEGMA v.2.3 (CEGMA, RRID:SCR_015055) analysis [15] suggested that 96.72% of CEGs could be found in the *A. monticola* assembly results, which is comparable to that of their corresponding ancestor with 98.69% (Supplementary Table S3b).

Besides the normal BUSCO [14] and CEG [15] estimation, transcriptome data of *A. monticola* can also be used for genome completeness assessment. We assembled the 11.96-Gb pooled transcriptome data from root, stem, leaf, flower, and seed of *A. monticola* into unigenes using Trinity v2.1.1 (Trinity, RRID:SCR_013048) [33] (Supplementary Table S3c). We also collected unigenes of *A. hypogaea* that generated from developmental transcriptome map [34]. We finally obtained 44,205 unigenes whose lengths are >500 bp (Supplementary Table S3d). Of which, 43,961 (99.45%) could be supported by the assembly results.

The completeness of the genome assembly was revealed by sequenced bases aligned along the entire length of the assembly. We remapped the Illumina short reads, RNAseq data, and PacBio subreads to the assembly results of *A. monticola*, respectively. For Illunima short reads and RNAseq data, we aligned paired-end reads to the genome of *A. monticola* by bwa-mem of BWA v0.7.10 (BWA, RRID:SCR_010910) [20] and found that more than 98.47% and 92.21% of them could be correctly remapped to assembly results, respectively (Supplementary Table S3e). We then remapped the error correction SMRT molecules from genome assembly data to assembly results of *A. monticola* by blasr v5.3 [35] and found that 92.16% of subreads had best alignments in assembly results (Supplementary Table S3e).

To evaluate the genome accuracy, we also randomly selected 20 SMRT molecules longer than 45 kb and aligned them to genome sequence. The coverage and identity of all molecules were >99% and 91%, respectively (Supplementary Table S3f). Additionally, the genome-wide Hi-C heatmap of *A. monticola*, shown by HiCplotter at 500-kb resolution, exhibited as expected that the frequency of intra-chromosome interactions rapidly decreased with linear distance (Fig. 3A). From the same Hi-C data, a similar genome-wide interaction map was observed for its ancestors *A. ipaensis* and *A. duranensis* (Fig. 3B). These comparison analyses suggested the high accuracy of *A. monticola* assemblies.

The assembly results achieved a high level of contiguity and connectivity for SMRT molecules, Illumina data, BioNano-genome map, and Hi-C data based on hybrid assembly of allotetraploid wild peanut genome. More than 91.83% of the assemblies were in ordered orientation in 20 pseudomolecules of two subgenomes, ranging from 39.68 Mb to 163.85 Mb (Table 1; Fig. 3A). The remaining 8.17% of the genome assembly was contained in 3,217 smaller scaffolds of at least 10 kb.

## Discussion

*A. monticola*(AABB-type genome, 2n = 4x = 40) is the only allotetraploid wild peanut within section *Arachis* and is regarded either as the direct progenitor of the cultivated peanut or as an introgressive derivative between the peanut and wild species [36, 37]. It is compatible with cultivated peanut in breeding, whereas its wild-type structure of fruits supports the maintenance of *A. monticola* as a separate taxonomic species [6, 38]. The generation of whole genome assemblies for *A. monticola* will provide a basis for the analysis of these interesting events among the genus *Arachis* during selection and/or domestication.

**Figure 2:**
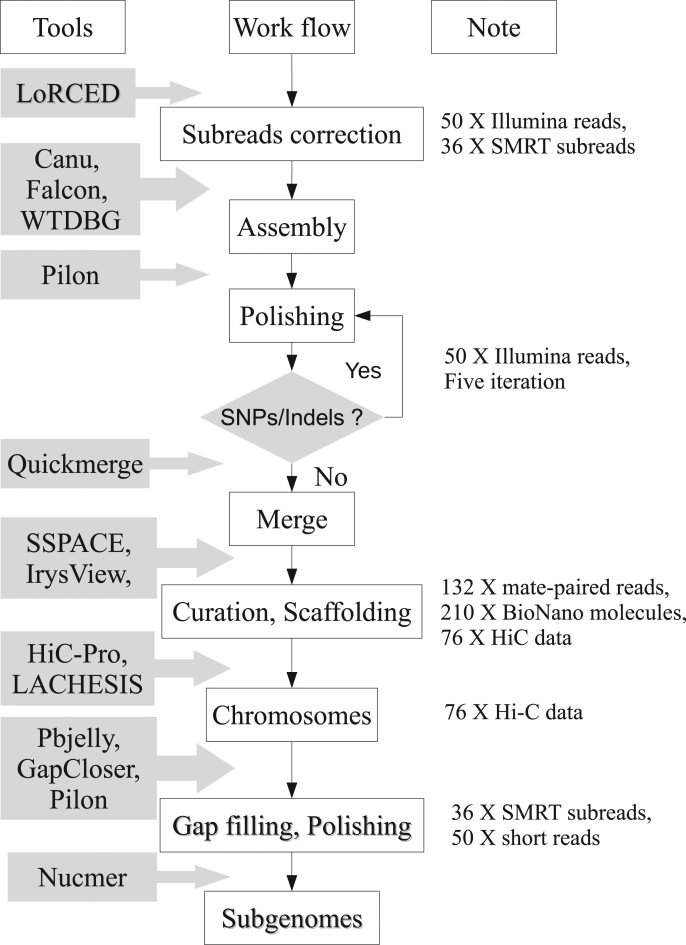
Work flow of assembly of alloteraploid wild peanut (*A. monticola*). We first corrected SMRT subreads by error correction module of Canu based on 36.10x Pacbio subreads. For subreads aborted by Canu, we corrected them with LoRDEC based on ∼50-fold coverage of Illumina short reads. Then we assembled these high-quality data using Canu, Falcon, and WTDGB, respectively, and used Pilon to polish them. To integrate advantages of different algorithms, we merged the assemblies by Quickmerge. We also curated “chimeric error” of genome assembly combing Pacbio molecules, BioNano data, and HiC links and scaffolded the contigs using SSPACE and IrysView. Further analysis of scaffold order and orientation through HiC-pro and LACHESIS led to chromosome-length scaffolds. SMRT subreads and short reads were used for gap filling and genome polishing through Pbjelly, GapCloser, and Pilon packages. The subgenomes of AA- and BB- genotypes were simply distinguished by the overall macro-synteny between genome assemblies and its corresponding ancestors.

We sequenced 171.44-fold genome coverage of a wild genotype, *A. monticola*, from 11 Illumina PE and MP libraries, ranging from 200-bp to 17-kb fragments (Supplementary Table S1a). A total of ∼462.87 Gb short reads enabled us to assemble a 1.996-Gb *A. monticola* genome (Supplementary Table S2a). We also generated a 36-fold sequencing coverage of *A. monticola* genome using 30 SMRT cells on the PacBio RS II and Sequel platforms (Supplementary Table S1b). Production of 11.5 million very long reads allowed us to generate a genome assembly that captures 2.24 Gb in 29,454 contigs (Supplementary Table S2c). We first assembled these contigs based on unique MP links of mapping results. The sequence number is significantly reduced from 29,454 contigs to 9,157 scaffolds, and the scaffold N50 improved from 120.61 kb to 491.06 kb (Supplementary Table S2c). To place these assemblies on super-scaffolds and determine the physical length of gaps between them, we developed a robust physical map from 2.88 million (∼596.26 Gb) high-quality BioNano optical molecules (Supplementary Table S1c). The assembles and N50 size of genome map is 2.65 Gb and 3.40 Mb, respectively, consisting of 1,404 sequences (Supplementary Table S1c). After genome curation of integrated evidence and hybrid assembly of assemblies and genome optical maps, we generated 2.62 Gb assembles, occupying 97.03% of the estimated genome size (Supplementary Table S2c). Adopting chromatin interaction mapping (Hi-C) links, we built the sequences of the 20 pseudomolecules that anchored 91.83% of the genome content (Fig. 2, Table 1). Referencing to the syntenic relationship between the sequences of *A. monticola* and those of its progenitors (*A. duranensis*, *A. ipaensis*), 96.07% of assemblies was successfully distinguished into two subgenomes (Table 1, Supplementary Figs S1 and S2).

Here we demonstrate the current state of the art for the *de novo* assembly of a highly complex genome for the allotetraploid wild peanut (*A. monticola*) based on long reads for contig formation, short reads for consensus validation, and scaffolding by MP links, optical map, and chromatin interaction mapping. These combined technologies produced reference-quality genome of tetraploid wild peanut, with chromosome-length scaffolds (Table 1, Supplementary Table S2b). Our assemblies represented a 5-fold improvement in continuity attributing to properly assembled gaps compared to the previously published *A. duranensis* and *A. ipaensis* assembly and better resolved the repetitive structures longer than 10 kb, especially the nearly identical regions of the two subgenomes (Table 2, Supplementary Table S2d).

**Figure 3:**
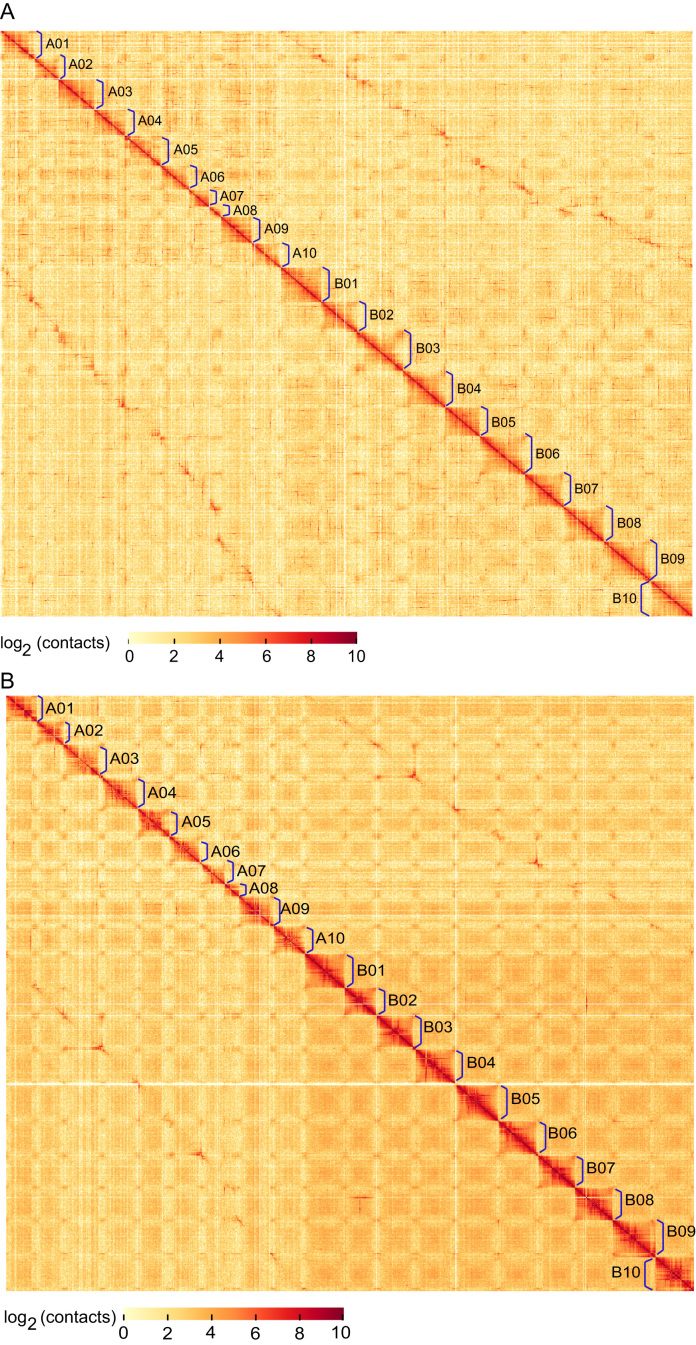
Interaction frequency distribution of Hi-C links among chromosomes. (**A**) Genome-wide Hi-C map of *A. monticola*. (**B**) Genome-wide Hi-C map of *A. ipaensis* and *A. duranensis*. We scanned the genome by 500-kb nonoverlapping window as a bin and calculated valid interaction links of Hi-C data between any pair of bins. The log2 of link number was calculated. The distribution of links among chromosomes was exhibited by heatmap based on HiCplotter. The color key of heatmap ranging from light yellow to dark red indicated the frequency of Hi-C interaction links from low to high (0∼10).

Taken together, we have developed an integrated approach, including "whole-genome sequencing and Pacbio and BioNano optics and Hi-C” to the sequencing and assembly of an allopolyploid *A. monticola* genome (Fig. 2). The final assembly comprised of 28,581contigs (N50 = 129.50 kb) and 4,135 scaffolds (N50 = 118.65 Mb) (Supplementary Table S2c) and can be organized into 20 chromosomes, including 1.06 Gb in the A subgenome and 1.45 Gb in the B subgenome (Table 1; Fig. 3A). Our assembly contains 97% of the *A. monticola* genome sequence.

The *A. monticola* genome presented here provides, for the first time, a reference genome for future studies of this important tetraploid wild peanut, which may be the “bridge” connecting the diploid wild species and tetraploid cultivated species to study subgenomes evolution, origin, and domestication among *Arachis* genus and other plants and will provide a wealth of information to enable studies of phylogeny, genome duplication, and convergent evolution [39]. The atlas data of the *A. monticola* genome will provide a valuable resource and facilitate future functional genomics and molecular-assisted breeding in this oil crop. Meanwhile, more reference information should be beneficial for studying the genetic changes during the recent polyploidization event and producing more elite peanut cultivars.

## Availability of supporting data

The Whole Genome Shotgun project has been deposited at DDBJ/ENA/GenBank under the accession QBTX00000000. The version described in this paper is version QBTX01000000. Raw reads of SMRT, WGS and Hi-C, and *A. monticola* genome assembly sequences of the*A. monticola* genome project have been deposited at the NCBI GeneBank under BioProject PRJNA430760 and BioSample Accession SAMN08378480. All supplementary figures and tables are provided in Additional Files. Supporting data including annotations and RNA-seq data are also available in the GigaDB database [40].

## Additional files

**Table S1a**. Summary of Illumina data for *A. monticola*.

**Table S1b**. Statistic of PacBio subreads length distribution for *A. monticola*.

**Table S1c**. Summary of BioNano data collection and assembly statistics.

**Table S1d**. Summary of HiC data for error correction and chromosome construction.

**Table S2a**. Summary of assembly results from Illumina short reads.

**Table S2b**. Summary of assembly results of different tools for *A. monticola*.

**Table S2c**. Summary of assembly results of different versions for *A. monticola*.

**Table S2d**. Comparison of genome assembly between *A. monticola* and corresponding ancestors *A. duranensis* and *A. ipaensis*.

**Table S3a**. Genome completeness assessment by BUSCO.

**Table S3b**. Completeness analysis based on CEG database.

**Table S3c**. Summary of pooled transcriptome data assisted for genome annotation

**Table S3d**. Genome completeness evaluated by ESTs/unigenes.

**Table S3e**. Genome completeness assessment based on sequencing reads.

**Table S3f**. PacBio sub-reads validation for the *A. monticola* genome assembly.

**Figure S1**. Circos plot showing shared synteny between *A. monticola* and *A. duranensis*.

**Figure S2**. Circos plot showing shared synteny between *A. monticola* and *A. ipaensis*.

## Abbreviations

BUSCO: Benchmarking Universal Single-Copy Orthologs; CEGMA: core eukaryotic gene-mapping approach; Gb: gigabase; Hi-C: high-throughput chromosome conformation capture; Kb: kilobase; Mb: megabase; PacBio: Pacific Biosciences; PE: paired-end; SMRT: single molecule real-time sequencing.

## Competing interests

The authors declare that they have no competing interests.

## Supplementary Material

GIGA-D-18-00025_Original_Submission.pdf

GIGA-D-18-00025_Revision_1.pdf

GIGA-D-18-00025_Revision_2.pdf

GIGA-D-18-00025_Revision_3.pdf

Response_to_Reviewer_Comments_Original_Submission.pdf

Response_to_Reviewer_Comments_Revision_1.pdf

Response_to_Reviewer_Comments_Revision_2.pdf

Reviewer_1_Report_(Original_Submission) -- Steven Cannon 2/13/2018 Reviewed

Reviewer_1_Report_(Revision_1) -- Steven Cannon 4/4/2018 Reviewed

Reviewer_1_Report_(Revision_2) -- Steven Cannon 4/29/2018 Reviewed

Reviewer_2_Report_(Original_Submission) -- Manish Kumar Pandey, Ph.D. 2/23/2018 Reviewed

Supplement Files
