# Supplementary material for: Genome of an allotetraploid wild peanut Arachis monticola: a de novo assembly
Source: Gigascience. 2018 Jun 19;7(6):giy066. doi: 10.1093/gigascience/giy066 (PMC6009596; doi:10.1093/gigascience/giy066)
Supplement: GIGA-D-18-00025_Revision_1.pdf [file giy066_giga-d-18-00025_revision_1.pdf]

GigaScience

Genome of an allotetraploid wild peanut Arachis monticola: a de novo assembly

--Manuscript Draft--

**Manuscript Number:**	GIGA-D-18-00025R1
**Full Title:**	Genome of an allotetraploid wild peanut Arachis monticola: a de novo assembly
**Article Type:**	Data Note
**Funding Information:**	National Natural Science Foundation of China (31471525) — Dr. Dongmei Yin<br>Key program of NSFC-Henan United Fund (U1704232) — Dr. Dongmei Yin<br>key scientific and technological project in Henan Province (161100111000;S2012-05-G03)) — Dr. Dongmei Yin<br>Innovation Scientists and Technicians Troop Construction Projects of Henan Province (2018JR0001) — Dr. Dongmei Yin
**Abstract:**	Arachis monticola (2n = 4x = 40) is the only allotetraploid wild peanut within section Arachis, with an AABB-type genome of about ~2.7 Gb. The AA-type subgenome is derived from diploid wild peanut Arachis duranensis, and the BB-type subgenome is derived from diploid wild peanut Arachis ipaensis. A. monticola is regarded either as the direct progenitor of the cultivated peanut or as an introgressive derivative between the cultivated peanut and wild species. The large polyploidy genome structure and enormous nearly identical regions of the genome make the assembly of chromosomal pseudomolecules very challenging. Here we report the first reference quality assembly of A. momticola genome, using a series of advanced technologies. The final whole genome of A. monticola is ~2.62 Gb representing 97.04% of the estimated genome size, and has a contig N50 and scaffold N50 of 106.66 Kb and 124.92 Mb, respectively. The vast majority (91.83%) of the assembled sequence was anchored onto the 20 pseudo-chromosomes and 96.07% of assemblies were accurately separated into AA- and BB- subgenomes. We demonstrated efficiency of the current state of the strategy for de novo assembly of the highly complex allotetraploid species, wild peanut (A. monticola), based on whole-genome shotgun sequencing, single molecule real-time (SMRT) sequencing, high-throughput chromosome conformation capture (Hi-C) technology and BioNano optical genome map. These combined technologies produced reference-quality genome of the allotetraploid wild peanut, which is valuable for understanding the peanut domestication and evolution within Arachis genus and among legume crops.
**Corresponding Author:**	Dongmei Yin<br><br>CHINA
**Corresponding Author Secondary Information:**	
**Corresponding Author's Institution:**	
**Corresponding Author's Secondary Institution:**	
**First Author:**	Dongmei Yin
**First Author Secondary Information:**	
**Order of Authors:**	Dongmei Yin<br>Changmian Ji<br>Xingli Ma<br>Hang Li

Powered by Editorial Manager® and ProduXion Manager® from Aries Systems Corporation

	Wanke Zhang
	Song Li
	Fuyan liu
	Kunkun Zhao
	Fapeng Li
	Ke Li
	Longlong Ning
	Jialin He
	Yuejun Wang
	Fei Zhao
	Yilin Xie
	Hongkun Zheng
	Xinguo Zhang
	Yijing Zhang
	Jinsong Zhang
**Order of Authors Secondary Information:**	
**Response to Reviewers:**	Reviewer #1: This paper reports what appears to be a high-quality genome assembly of the wild tetraploid peanut relative, Arachis monticola. The paper primarily reports the assembly methods and statistics. As far as I am able to tell, the assembly appears to have been made using state-of-the-art methods.<br><br>The main weakness (and a very large one) is that the assembly is not accessible. I assume it will be made available after embargo, but as this paper primarily describes the assembly resource, I don't feel that I can adequately assess the resource if it is not available. It appears that the accession IDs provided for GigaDB and GenBank are placeholders. I believe the standard practice is instead to provide actual identifiers provided by those databases - which may be held pending embargo release, depending on release conditions.<br><br>Response: we have provided the correct accessions in NCBI and GigaDB including project accession, biosample accession, SRA accessions of Illumina, pacbio and HiC data, and genome sequences accession in Availability of supporting data.<br><br>It would also have been nice for the authors to have provided some scientific analysis of the assembly, beyond a simple description of the resource. For example, no report is provided regarding sequence interactions (exchanges) between the subgenomes, or evidence about rearrangements in the progenitor species - or sequence similarity with the diploid progenitors.<br><br>Response: We appreciate your valuable suggestion. In this study, we released the genome of an allotetraploid wild peanut firstly for publication in Gigascience as “DATA NOTE” article, and we provided here the circus plot to demonstrate the high consistency of A and B subgenomes in tetraploid peanut with diploid ancestors (A. ipaensis and A. duranensis) (Sup Figure1-2). More detailed analysis are already under way and an interesting story will show in the following paper.<br><br>Specific comments:<br>Introduction<br>60: "has a key role on food security within the global society" -- better as "has a key role in food security globally" (on -> in and global society -> globally)<br><br>62: "The genus Arachis is unique of subterranean fruit originated" --> The genus Arachis is unique for its subterranean fruit, which originates from

Powered by Editorial Manager® and ProduXion Manager® from Aries Systems Corporation

82: "billion basesand" --> : billion bases and

84: occupying --> spanning

Response: we have revised the MS following the advises in line 61, 62, 99 and 101.

Availability of supporting data:
354: At http://gigadb.org, I find no record for RRID:SCR00000 or for SCR00000. This seems to be a placeholder. The correct accession will be needed.

Response: we have revised the MS following the advises in line 388 (Availability of supporting data).

355: "Raw data were deposited in the Sequence Read Archive (SRA00000) for the Arachis monticola genome." -- the accession SRA00000 seems to be a placeholder. At GenBank, I see biosample SAMN05572396 and bioproject PRJNA248910 for Arachis monticola, but from a different research group. The correct accession(s) will be needed.

Response: we have provided the correct accessions in NCBI and GigaDB including project accession, biosample accession, SRA accessions of Illumina, pacbio and HiC data, and genome sequences accession in Availability of supporting data.

Reviewer #2: The genome assembly for Arachis monticola is very important for Arachis species research and therefore peanut research will be highly benefited by this resource. I have following comments for the authors:
1. Needs proper justification that why A. monticola is so important for peanut improvement as this wild relative of cultivated peanut

Response: We agree with this comment and associated content has been added in the revised manuscript (line 62~73, 82~91).

2. Details on the genotype/accession not provided throughout MS not provided

Response: We have added the genotype/accession of the sequencing samples in MS, from line 105 to 107.

3. The information provided in the first para of Results section was again repeated in detail in rest of the sections of the Results...looked much text repetitive

Response: We have rewrote the Results and removed the redundant contents from line 90~102 of previous version to line 113~117 of revised version.

4. The major problem with this MS is that this MS appears totally descriptive in nature and no scientific advancement been provided to the available understanding/knowledge

Response: We have revised the MS as review’s advises from line 62~73, 82~91 in the revised version.

5. Sequencing genome is not just enough for publications..until unless it makes some sense for the research community

Response: We follow the review’s points. Although previous publication of reference genome sequences of peanut diploid ancestors (A. ipaensis and A. duranensis) provide the valuable understanding and knowledge of peanut/legume and facilitated the peanut research, all the cultivated peanut varieties are allotetraploids. The high quality reference genome of allotetraploid peanut is important for evolution, origin and domestication research of wild and cultivated peanuts, and favorable to peanut breeding, which is helpful for peanut research community. So we published the genome assembly as data note as soon as possible, which would benefit to community and scientists. The further research including subgenomes evolution, origin and domestication also have been planned. Here, we provide the circus plot to

Powered by Editorial Manager® and ProduXion Manager® from Aries Systems Corporation

	demonstrate the high consistency of A and B subgenomes in tetraploid peanut with diploid ancestors (A. ipaensis and A. duranensis).<br><br>6. The MS in present form does not add value to much for peanut research community except providing genome assembly of A. monticola for research<br><br>Response: Please refer to the response to comment 5.<br><br>7. Authors are suggested to include some evolutionary, origin and domestication related analysis and results for improving this MS<br><br>Response: Please refer to the response to comment 5.
**Additional Information:**	
**Question**	**Response**
Are you submitting this manuscript to a special series or article collection?	No
**Experimental design and statistics**<br><br>Full details of the experimental design and statistical methods used should be given in the Methods section, as detailed in our Minimum Standards Reporting Checklist. Information essential to interpreting the data presented should be made available in the figure legends.<br><br>Have you included all the information requested in your manuscript?	Yes
**Resources**<br><br>A description of all resources used, including antibodies, cell lines, animals and software tools, with enough information to allow them to be uniquely identified, should be included in the Methods section. Authors are strongly encouraged to cite Research Resource Identifiers (RRIDs) for antibodies, model organisms and tools, where possible.<br><br>Have you included the information requested as detailed in our Minimum Standards Reporting Checklist?	Yes
**Availability of data and materials**<br><br>All datasets and code on which the conclusions of the paper rely must be either included in your submission or deposited in publicly available repositories (where available and ethically appropriate), referencing such data using	Yes

Powered by Editorial Manager® and ProduXion Manager® from Aries Systems Corporation

a unique identifier in the references and in the “Availability of Data and Materials” section of your manuscript.<br><br>Have you have met the above requirement as detailed in our Minimum Standards Reporting Checklist?	

Manuscript

Click here to download Manuscript Genome of an allotetraploid wild peanut _G_revised 2018 0312.doc

DATA NOTE

Genome of an allotetraploid wild peanut *Arachis monticola*: a de novo assembly

Dongmei Yin [1]*†, Changmian Ji [2,]†, Xingli Ma[1,]†, Hang Li[2], Wanke Zhang[3], Song Li[2], Fuyan Liu[2], Kunkun Zhao[1], Fapeng Li[1], Ke Li[1], Longlong Ning[1], Jialin He[1], Yuejun Wang[4], Fei Zhao[4], Yilin Xie[4], Hongkun Zheng[2], Xingguo Zhang[1]*, Yijing Zhang[4]*, Jinsong Zhang[3]*

1 College of Agronomy, Henan Agricultural University, Zhengzhou 450002, China

2 Biomarker Technologies Corporation, Beijing 101300, China

3 State Key Lab of Plant Genomics, Institute of Genetics and Developmental Biology, Chinese Academy of Sciences, Beijing 100101, China

4 National Key Laboratory of Plant Molecular Genetics, CAS Center for Excellence in Molecular Plant Sciences, Institute of Plant Physiology and Ecology, Shanghai Institutes for Biological Sciences, Chinese Academy of Sciences, Shanghai 200032, China

*Corresponding author. Dongmei Yin, Xingguo Zhang, College of Agronomy, Henan Agricultural University, Rd. Wenhua No. 95, Zhengzhou, Henan 450002, P. R. China. Tel: 0086-371-63558122; E-mail: yindm@126.com; hnndzxg@126.com; Jinsong Zhang, State Key Lab of Plant Genomics, Institute of Genetics and Developmental Biology, Chinese Academy of Sciences, Beijing 100101, P. R. China. Tel: 0086-10-64807601; E-mail: jszhang@gentics.ac.cn; Yijing Zhang, Institute of Plant Physiology and Ecology, Shanghai Institutes for Biological Sciences, Chinese Academy of Sciences, Shanghai 200032, P. R.China. Tel: 86-21-54924204; Email: zhangyijing@sibs.ac.cn

† Equal contribution

Abstract

Arachis monticola (2n = 4x = 40) is the only allotetraploid wild peanut within section *Arachis*, with an AABB-type genome of about ~2.7 Gb. The AA-type subgenome is derived from diploid wild peanut *Arachis duranensis*, and the BB-type subgenome is derived from diploid wild peanut *Arachis ipaensis*. *A. monticola* is regarded either as the direct progenitor of the cultivated peanut or as an introgressive derivative between the cultivated peanut and wild species. The large polyploidy genome structure and enormous nearly identical regions of the genome make the assembly of chromosomal pseudomolecules very challenging. Here we report the first reference quality assembly of *A. momticola* genome, using a series of advanced technologies. The final whole genome of *A. monticola* is ~2.62 Gb representing 97.04% of the estimated genome size, and has a contig N50 and scaffold N50 of 106.66 Kb and 124.92 Mb, respectively. The vast majority (91.83%) of the assembled sequence was anchored onto the 20 pseudo-chromosomes and 96.07% of assemblies were accurately separated into AA- and BB- subgenomes. We demonstrated efficiency of the current state of the strategy for de novo assembly of the highly complex allotetraploid species, wild peanut (*A. monticola*), based on whole-genome shotgun sequencing, single molecule real-time (SMRT) sequencing, high-throughput chromosome conformation capture (Hi-C) technology and BioNano optical genome map. These combined technologies produced reference-quality genome of the allotetraploid wild peanut, which is valuable for understanding the peanut domestication and evolution within *Arachis* genus and among legume crops.

Introduction

Peanut (*Arachis hypogaea* L.) is widely cultivated in subtropical and tropical regions as a plant-based resource for protein and edible oil, which has a key role in food security globally. The genus *Arachis* is unique for its subterranean fruit, which is originated in South America and has ~80 described species divided into nine sections based on their morphology, cross compatibility relationships and geographical distribution [1]. Section *Arachis* is of particular interest because it contains 30 diploid wildspecies, one tetraploid wild species (*A. monticola*) and cultivated peanut (*A. hypogaea*) (2n=4x=40). *A. monticola* was identical to the accession of *A. hypogaea* with high genetic identity [2, 3]. Moreover, hybrids between *A. monticola* and *A. hypogaea* are fertile [4]. *A. monticola* is considered a distinct species from *A. hypogaea* based mainly on its fruit structure, which has an isthmus separating each seed, resembling the diploid wild species [5]. Comparison of the genomes among the *A. monticola*, *A. hypogaea* and wild species should shed light on the evolutionary and/or domesticated events undergoing in the cultivated species.

As a relatively young allotetraploid species, the genome of wild peanut *A. monticola* exhibits complexity with an AABB-type genome of about ~2.7 Gb [6] and shares many regions of high similarity between its two subgenomes [7]. Challenges are present for its genome assembly due to the large polyploidy genome structure and highly homologous genomic sequences. Because of these difficulties, sequencing of the diploid ancestors *A.ipaensis* and *A.duranensis* was first completed [7]. The total

assembled genome sizes were 1.025 Gb and 1.338 Gb respectively for the two species, with an N50 contig length of 22 Kb, using paired-end Illumina sequencing. All *A.ipaensis* pseudomolecules were larger than their *A.duranensis* counterparts and *A.ipaensis* may be a direct descendant which contributed the B subgenome to cultivated peanut [7]. Although previous publication of reference genome sequences of peanut diploid ancestors (*A. ipaensis* and *A. duranensis*) provide the valuable understanding and knowledge of peanut/legume and facilitated the peanut research, all the cultivated peanut varieties are allotetraploids. The high quality reference genome of allotetraploid peanut is important for evolution, origin and domestication research of wild and cultivated peanuts, and favorable to peanut breeding, which is helpful for peanut research community.

In this study, we used a series of advanced technologies, including whole-genome shotgun sequencing, single molecule real-time (SMRT) sequencing, high-throughput chromosome conformation capture (Hi-C) technology and BioNano optical genome mapping, to generate a high quality genome sequence for the tetroploid wild peanut species *A. monticola*. By combining these very long reads with highly accurate short reads, we have been able to produce an assembly of this tetroploid wild species (*A. monticola*) genome. In total, we used 767.25 billion bases and 210.83 fold genome coverage of BioNano data for the genome assembly. Finally, we generated 2.62 Gb assembles, spanning 97.04 % of the estimated genome size for *A. monticola*.

Results

Arachis monticola is an allotetraploid wild peanut species and has features different from the tetraploid cultivated peanut (Fig. 1). We selected the seeds of PI263393 lines for genome sequencing. The peanut plants were grown in growth chamber with 25 °C, and DNA was extracted from fresh leaves of 30 days old wild peanut seedlings. To create the *Arachis monticola* genome assembly, we generated four extremely large primary data sets including 462.87 Gb Illumina reads (Sup table 1a), 11.5 million SMRT long reads as ~91.71 Gb (Sup table 1b), 2.88 million (~596.26 Gb) high quality BioNano optical molecules (Sup table 1c) and 76.54-fold coverage of the genome of Hi-C data (Sup table 1d). All the reads were generated from the same *A.monticola* line. Take advantage of integrated technologies, we achieved 2.62 Gb high quality reference genome of wild peanut with 20 pseudo-chromosomes (Table 1 and Sup table 2c), and successfully distinguished two subgenomes (*A. mon-A* and *A.mon-B*) , corresponding to its diploid progenitors *A.ipaensis* and *A.duranensis*, respectively (Sup table 2d).

Initial genome assembly

An independent WGS assembly was executed using Allpath-LG v1.4 (Allpath-LG, RRID:SCR_010742) [8] to increase the lengths of scaffolds and to fill gaps in the *A. monticola* assembly. Eleven paired-end and mate-paired libraries ranging from 200 bp to 17 Kb were constructed and sequenced (Sup table 1a). From 171 fold coverage reads (~462.87 Gb), we assembled into 1.66 Gb results with scaffold N50 and contig

N50 of 369.06 Kb and 16.17 Kb, respectively (Sup table 2a).

We also assembled the *A. monticola* genome using 97.71 Gb long Pacific Biosciences (PacBio) reads, covering approximately 36.10 fold coverage of genome size (Sup table 1b). Because of a high error rate of PacBio reads, we first corrected these by error correction module of Canu v1.5 [9] based on 36.10 x Pacbio subreads. For subreads aborted by Canu, we corrected them with LoRDEC v0.5 [10] based on ~50 fold coverage of Illumina short reads. Finally, we retained 34.07 fold coverage of high quality subreads (92.78 Gb) and independently assembled them with Falcon v0.7 [11], WTDBG v1.2.8 [12] and Canu v1.5 [9]. The assembled size from Falcon, WTGDB and Canu are 1.88 Gb, 1.96 Gb and 2.26 Gb, respectively. The contig N50 of assembly results is 81.5 Kb, 82.8 Kb and 109.2 Kb, respectively for the three methods (Sup table 2b). The completeness assessment of these assembles through Benchmarking Universal Single-Copy Orthologs (BUSCO) databases (BUSCO, RRID:SCR_015008) [13] and Core Eukaryotic Genes Mapping Approach (CEGMA， RRID:SCR_015055) [14] showed that more than 96% CEGs and 90% of complete BUSCOs are detectable, suggesting the high completeness of the assembly results. We then polished the consensus sequence of three assemblies based on 50 x Illumina pair-end reads using Pilon v1.22 software [15]. To take advantage of assemblies from different tools and generate more contiguity and connectivity results, we merged them together with quickmerge v0.2 package [16]. The strict conditions were considered in this step to avoid chimeric errors. We obtained a genome of 2.24 Gb with contig N50 and longest contig of 120.61 Kb and 1.89 Mb, respectively (Sup table 2b).

Physical map construction

To develop a robust physical map for the allotetraploid wild peanut that could be helpful to place sequence contigs on chromosomes and to determine the physical length of gaps between them [17], we constructed BioNano optical genome map libraries for the sequencing genotype from the fresh leaves. From the enzyme density and ditribution assessment of genome sequences using Label Density Calculator v1.3.0 (BioNano Genomics, CA, US), we adopted the Nt.BspQI nickase for optical map library construction. The basic process of BioNano raw data was conducted using IrysView v2.5.1 package (BioNano Genomics, CA, US). Molecules whose lengths are more than 150 kb (with label SNR >= 3.0 and average molecule intensity < 0.6) were retained for further genome assembling. We obtained 2.8 million (~596.26 Gb) high quality optical molecules, accounting for ~210 x coverage of genome size (Sup table 1c). The N50 of the molecules is 210.83 Kb (Sup table 1c). On the basis of the label positions on single DNA molecules, de novo assembly was performed by a pairwise comparison of all single molecules and overlap-layout-consensus path building, which adopted by IrysView v2.5.1 assembler [18]. The parameter set for large genomes was used for assembly with the IrysView software. We considered only molecules containing more than seven nicking enzyme sites for assembly (min label per molecule: 8). A *p* value threshold of 1e-8 was used during the pairwise assembly, and 1e-9 for extension and refinement steps and 1e-12 for merging contigs were adopted. The resulting physical map covers approximately 2.65 Gb (around 98.15%

of the 2.7 Gb genome size). We generated 1,404 optical map-based scaffolds with N50 of 3.4 Mb for *A. monticola* (Sup table 1c). The high quality optical map would be used for genome curation and hybrid assembly with SMRT-based assembly, combing the MP links and Hi-C data.

Scaffold construction and curation

Total of nine mate-pair libraries ranging from 3 kb to 17 kb fragments were prepared for scaffolds, which accounted for ~132 fold coverage of previous estimated genome size (2.7 Gb) [6] (Sup table 1a). To decrease the chimeric in initial assembly results, we mapped the different fragment mate-paired data to the contigs using BWA v0.7.10 (BWA, RRID:SCR_010910) [19], considering only unique mapping reads for further scaffolds construction. Further scaffolding was performed by SSPACE v3.0 (SSPACE, RRID:SCR_005056) [20]. Contigs are assembled into scaffolds with mate-pair (MP) information, estimating gaps between the contigs according to the distance of MP links. Two contigs supported by at least 3 reasonable MP links in each fragment libraries (insert size +- 5SD) were joined as a scaffold. We assembled 29,454 contigs into 9,157 scaffolds with large reasonable intra-gaps sequences (Sup table 2c). In this step, we obtained 2.35 Gb assembly results for *A. monticola*, whose scaffold N50 and L50 are 491.06 Kb and 1,396, respectively (Sup table 2c).

As a relatively young allotetraploid species, the genome of *A. monticola* is particularly complicated especially considering the phenomenon of partially homologous sequences between its two subgenomes [7, 21]. The assembly results of

allotetraploid genome from SMRT reads may introduce lots of chimeric errors from high homologous and/or large repeated regions of *A.monticola*. The optical map of single molecules from BioNano Genomics' Irys® System could assemble large homologous and repeated regions, taking advantage of its super long molecule reads. As a result, detection of conflicts between contigs/scaffolds and genome map, and correction of the potential errors are strongly necessary and feasible.

To ascertain the quality of assembly results, we generated an *in silico* map of merged results by Knickers v1.5.5.0 program [18] with Nt.BspQI nickase. From the comparison between the contigs/scaffolds and optical maps by RefAligner v5122 [18], we identified 610 conflicts. Next Generation Mapping (NGM-HS) was used to resolve conflicts between the sequence and optical map assemblies by breaking conflict point of assembly. Conflicts were identified based on chimeric score of a conflict junction, mate-pairs information and SMRT molecules alignment result, which is near the conflict junctions on the optical genome map. The chimeric score of conflict junction is defined as the percentage of BioNano molecules that fully align to the 50 kb flanks of optical map. If the chimeric scores of the conflict junction were ≥ 30, and more than two fully aligned optical molecules located across the conflict junction of genome map, we suggested a candidate chimerical error in scaffold/contig sequence. The alignment results of conflict regions were visualized in IrysView [18] for manual investigation. Knickers, RefAligner, and IrysView were obtained from BioNano Genomics [18]. Further investigation of mate-paired links and SMRT molecules alignment would assist to make a decision of cutting on selected sequences. If the

mate-pair relationship (3Kb~17Kb) of 10Kb flanks of conflict junction is in disagreement, or less than 5 coverage of fully aligned Pacbio molecules are across this region, we suggested breaking the point. We considered the consistent soft-clip sites of SMRT molecules on reference sequence as accurate break point. All proposed cuts were manually evaluated using BioNano molecule-to-genome map alignments, SMRT molecule-to-sequence contig alignments, and mate-paired libraries mapping results based on integrated graphic platform. Of these conflicts, 600 were chimeric in the long reads assembly, and 10 were left unresolved. After chimeric correction, we assembled the 6,262 hybrid scaffolds based on genome map hybrid assembly. The genome size of *A. monticola* is 2.62 Gb, with scaffold N50 of 1.51 Mb (Sup table 2c).

Gap filling and SMRT-error correction

To improve the contiguity of assembly results, we fulfilled the gap filling process combined SMRT sequencing data, Illumina data. PBJelly [22] was used to fill gaps using approximately 34.07 fold coverage of error- corrected SMRT sequencing data from initial genome assembly step. Then we further filled retaining gaps using 39 fold coverage pair-end data (Sup table 1a), along with *de Bruijn* graph analysis to detect instances where a unique path of reads spanned a gap, implemented with Gapcloser v1.12 of SOAPDenovo packages (GapCloser, RRID:SCR_015026) [23]. During the gap-filling procedure, 42.87 Mb gaps were filled by SMRT long reads and Illumina data.

To ensure base-pairing accuracy of assembly results from SMRT molecules, we further polished the consensus sequence after the construction of the pseudomolecules based on ~105 Gb Illumina pair-end reads using Pilon [15]. A total of 5,607 kb bases, including SNPs and small Indels, were corrected, of which 0.21% were small indels.

Pseudomolecules construction and sub-genome identification

High-throughput chromosome conformation capture (Hi-C) technology enables the generation of genome-wide 3D proximity maps and is an efficient and low-cost strategy for sequences cluster, ordered and orientation for pseudomolecule construction [24]. This technology has been successfully applied in recent complex genome projects including goat [25], Tartary buckwheat [26], wild emmer [27], and barely [28]. We constructed three Hi-C fragment libraries ranging from 300-700 bp and sequenced them through X-TEN platform for pseudomolecules construction. Mapping of Hi-C reads and assignment to restriction fragments were performed as described elsewhere [24]. Briefly, adapter sequences of raw reads were trimmed with cutadapt v1.0 (cutadapt, RRID:SCR_011841) [29] and low quality PE reads were removed for clean data. The clean Hi-C reads, accounting for ~ 60 fold coverage of *A. monticola* genome, were mapped to the assembly results with bwa align v0.7.10 (BWA, RRID:SCR_010910) [19] (Sup table 1d). Only uniquely aligned pairs read whose map quality is more than 20 were considered for further analysis. Duplicate removal, sorting and quality assessment were performed with HiC-Pro v2.8.1 [30]. The 21.98 % of Hi-C data was valid interaction pairs. Raw counts of Hi-C links were

aggregated in 50 kb bins and normalized separately for intra- and inter- chromosomal contacts using LACHESIS [24]. We clustered the sequences into initial 20 groups according to threshold of the contact frequency. For each group, we clustered the sequences in 5 subgroups and independently decided the order and orientation of sequences based on contact probability of each sub-groups. The whole order and orientation subgroup was considered as super-bin and recalculated for the interaction matrices for each group. Then LACHESIS [24] was used to assign the order and orientation of each group. Based on 76.54 fold coverage of Hi-C data, the vast majority (91.83%) of the assembled sequence was anchored onto the 20 pseudo-chromosomes by frequency distribution of valid interaction pairs (Table 1).

Benefited from the published genomes of *A. duranensi*s and *A. ipaensis*, the donors of alloteraploid peanut, we are able to directly identify the corresponding subgenomes based on the whole genome comparison between the assembly results of *A. monticola* and the two wild diploid peanuts. We aligned the assembly results to its ancestral genomes with Mummer v2.23 [31] and successfully distinguished more than 96.07% of sequences into *A.mon*-A and *A.mon*-B subgenomes (Table 1). Finally, the subgenome size of *A.mon* -A and *A.mon* -B is 1,035.76 Mb and 1,485.16 Mb, respectively, which is comparable to that of their ancestors *A. duranensi*s and *A. ipaensis* (Table 2; Sup table 2d).

Genome quality assessment

Completeness of gene-space representation was evaluated based on the plants dataset of the Benchmarking Universal Single-Copy Orthologs (BUSCO) database with the

BUSCO pipeline v3.0.2 (BUSCO, RRID:SCR_015008) [13]. The results showed that 91.67% of complete gene models could be detected in *A. monticola* genome (Sup table 3a). Comparison analysis suggested that the gene region completeness of assemblies is slightly better than their corresponding progenitors (Sup table 3a).

The core eukaryotic gene-mapping approach (CEGMA) [14] provides a simple method to rapidly assess genome completeness. It comprises a set of highly conserved, single-copy genes, present in all eukaryotes, including 458 core eukaryotic genes (CEGs) and 248 of which are highly conserved CEGs. CEGMA v.2.3 (CEGMA, RRID:SCR_015055) analysis [14] suggested that 96.72% of CEGs could be found in the *A. monticola* assembly results, which is comparable to that of their corresponding ancestor with 98.69% (Sup table 3b).

Besides the normal BUSCO [13] and CEGs [14] estimation, transcriptome data of *A. monticola* can also be used for genome completeness assessment. We assembled the 11.96 Gb pooled transcriptome data from root, stem, leaf, flower and seed of *A. monticola* into unigenes using Trinity v2.1.1 (Trinity, RRID:SCR_013048) [32] (Sup table 3c). We also collected unigenes of *A. hypogaea* which generated from developmental transcriptome map (https://www.peanutbase.org/download). We finally obtained 44,205 unigenes whose lengths are more than 500 bp (Sup table 3d). Of which, 43,961 (99.45%) could be supported by the assembly results.

The completeness of the genome assembly was revealed by sequenced bases from aligned along the entire length of the assembly. We remapped the Illumina short reads, RNAseq data and PacBio subreads to the assembly results of *A. monticola*,

respectively. For Illunima short reads and RNAseq data, we aligned paired-end reads to the genome of *A. monticola* by bwa-mem of BWA v0.7.10 (BWA, RRID:SCR_010910) [19] and found that more than 98.47% and 92.21% of them could be correctly remapped to assembly results, respectively (Sup table 3e). We then remapped the error correction SMRT molecules from genome assembly data to assembly results of *A. monticola* by blasr v5.3 [33] and found that 92.16% of subreads had best alignments in assembly results (Sup table 3e).

To evaluate the genome accuracy, we also randomly selected 20 SMRT molecules longer than 45 Kb and aligned to genome sequence. The coverage and identity of all molecules have greater than 99% and 91%, respectively (Sup table 3f). Additionally, the genome-wide Hi-C heatmap of *A. monticola* which shown by HiCplotter at 500 Kb resolution exhibited as expectation that the frequency of intra-chromosome interactions rapidly decrease with linear distance (Fig. 3A). From the same Hi-C data, similar genome-wide interactions map was observed for its ancestors *A. ipaensis* and *A. duranensis* (Fig. 3B). These comparison analysis suggested the high accuracy of *A. monticola* assemblies.

The assembly results achieved a high level of contiguity and connectivity for SMRT molecules, Illumina data, BioNano-genome map and Hi-C data based on hybrid assembly of allotetraploid wild peanut genome. More than 91.83 % of the assemblies were in ordered orientation in 20 pseudomolecules of two subgenomes, ranging from 39.68 Mb to 163.85 Mb (Table 1; Fig. 3A). The remaining 8.17% of the genome assembly was contained in 3,217 smaller scaffolds of at least 10 Kb.

Discussion

*Arachis monticola(*AABB-type genome, 2n = 4x = 40) is the only allotetraploid wild peanut within section *Arachis,* and is regarded either as the direct progenitor of the cultivated peanut or as an introgressive derivative between the peanut and wild species [34, 35]. It is compatible with cultivated peanut in breeding whereas its wild type structure of fruits supports the maintenance of *A. monticola* as a separate taxonomic species [36, 37]. The generation of whole genome assemblies for *A. monticola* will provide basis for the analysis of these interesting events among the genus *Arachis* during selection and/or domestication.

We sequenced 171.44-fold genome coverage of a wild genotype *A. monticola* from 11 Illumina paired-end (PE) and meta-paired (MP) libraries, ranging from 200 bp to 17 Kb fragments (Sup table 1a). A total of ~462.87 Gb short reads enabled us to assemble 1.996 Gb *A. monticola* genome (Sup table 2a). We also generated a 36 fold sequencing coverage of *A. monticola* genome using 30 single molecule real-time (SMRT) cells on the PacBio RS II and Sequal platforms (Sup table 1b). Production of 11.5 million very long reads allowed us to generate a genome assembly that captures 2.24 gigabases (Gb) in 29,454 contigs (Sup table 2c). We first assembled these contigs based on unique MP links of mapping results. The sequence number is significantly reduced from 29,454 contigs to 9,157 scaffolds, and the scaffold N50 is improved from 120.61 Kb to 491.06 Kb (Sup table 2c). To place these assemblies on super-scaffolds and determine the physical length of gaps between them, we

developed a robust physical map from 2.88 million (~596.26 Gb) high quality BioNano optical molecules (Sup table 1c). The assembles and N50 size of genome map is 2.65 Gb and 3.40 Mb, respectively, consisting of 1,404 sequences (Sup table 1c). After genome curation of integrated evidence and hybrid assembly of assemblies and genome optical map, we generated 2.62 Gb assembles, occupying 97.03% of the estimated genome size (Sup table 2c). Adopting chromatin interaction mapping (Hi-C) links, we build the sequences of the 20 pseudomolecules that anchored 91.83% of the genome content (Fig. 2 and Table 1). Referencing to the syntenic relationship between the sequences of *A.monticola* and those of its progenitors (*A. duranensis, A. ipaensis*), 96.07% of assemblies was successfully distinguished into two subgenomes (Table 1 and Sup figure 1- 2).

We demonstrated current state of the strategy for the de novo assembled highly complex genome for the allotetraploid wild peanut (*A. monticola*), based on long reads for contig formation, short reads for consensus validation, and scaffolding by MP links, optical map and chromatin interaction mapping. These combined technologies produced reference-quality genome of tetraploid wild peanut, with chromosome-length scaffolds (Table 1 and Sup table 2b). Our assemblies represented a five-fold improvement in continuity attributing to properly assembled gaps, compared to the previously published *A. duranensis* and *A. ipaensis* assembly, and better resolved the repetitive structures longer than 10 Kb, especially the nearly identical regions of the two subgenomes (Table 2 and Sup table 2d).

Taken together, we have developed an integrated approach, including "WGS and Pacbio and BioNano optics and Hi-C", to the sequencing and assembly of an allopolyploid *Arachis monticola* genome(Fig. 2). The final assembly comprised of 28,581contigs (N50=129.50 Kb) and 4,135 scaffolds (N50=118.65 Mb) (Sup table 2c), and can be organized into 20 chromosomes, including 1.06 Gb in the A subgenome and 1.45 Gb in the B subgenome (Table 1; Fig. 3A). Our assembly contains 97.03% of the *A. monticola* genome sequence.

The *Arachis monticola* genome presented here provides, for the first time, a reference genome for future studies of this important tetraploid wild peanut, which may be the "bridge" connecting the diploid wild species and tetraploid cultivated species to study subgenomes evolution, origin and domestication among *Arachis* genus and other plants which will provide a wealth of information to enable studies of phylogeny, genome duplication, and convergent evolution [38]. The atlas data of the *A. monticola* genome will provide a valuable resource and facilitate future functional genomics and molecular-assisted breeding in this oil crop. Meanwhile, more reference information should be beneficial for studying the genetic changes during the recent polyploidization event and producing more elite peanut cultivars.

Availability of supporting data

Raw reads, genome assembly sequences of *Arachis monticola* genome project have been deposited at the NCBI GeneBank under BioProject Accession PRJNA430760 and BioSample Accession SAMN08378480. All supplementary figures and tables are provided in Additional Files. Supporting data are also available in the GigaDB database (GigaDB, RRID:RRID:SCR_004002).

Additional files

Sup table 1a. Summary of illumina data for *A. monticola*.

Sup table 1b. Statistic of PacBio sub-reads length distribution for *A. monticola*.

Sup table 1c. Summary of BioNano data collection and assembly statistics.

Sup table 1d. Summary of HiC data for error correction and chromosome construction.

Sup table 2a. Summary of assembly results from Illumina short reads.

Sup table 2b. Summary of assembly results of different tools for *A. monticola*.

Sup table 2c. Summary of assembly results of different versions for *A. monticola*.

Sup table 2d. Comparison of genome assembly between *A. monticola* and corresponding ancestors *A.duranensis* and *A.ipaensis*.

Sup table 3a. Genome completeness assessment by BUSCO.

Sup table 3b. Completeness analysis based on CEG database.

Sup table 3c. Summary of pooled transcriptome data assisted for genome annotation.

Sup table 4c.Genome completeness assessment based on sequencing reads.

Sup table 3d. Genome completeness evaluated by ESTs/unigenes.

Sup table 3e.Genome completeness assessment based on sequencing reads.

Sup table 3f. PacBio sub-reads validation for *A. monticola* genome assembly.

Sup figure 1. Circus plot showing shared synteny between *A. monticola* and *A. duranensis.*

Sup figure 2. Circus plot showing shared synteny between *A. monticola* and *A.*

ipaensis.

Competing interests

The authors declare that they have no competing interests.

Acknowledgments

This work was financially supported by grants from the National Natural Science Foundation of China (No. 31471525); Key program of NSFC-Henan United Fund（No. U1704232）; key scientific and technological project in Henan Province (No.161100111000； S2012-05-G03); Innovation Scientists and Technicians Troop Construction Projects of Henan Province（No.2018JR0001）

Reference

1. Krapovickas, A. and W.C. Gregory, *Taxonomıía del género Arachis (Léeguminosae).* Bonplandia, 1994. **8**(1-4): p. 1-186.
2. Hilu, K.W. and H.T. Stalker, *Genetic relationships between peanut and wild species of Arachis sect. Arachis (Fabaceae): Evidence from RAPDs.* Plant Systematics & Evolution, 1995. **198**(3-4): p. 167-178.
3. Re, D., *Genetic diversity of cultivated and wild-type peanuts evaluated with M13-tailed SSR markers and sequencing.* Genetical Research, 2007. **89**(2): p. 93-106.
4. Pattee, H.E., H.T. Stalker, and F.G. Giesbrecht, *Reproductive Efficiency in Reciprocal Crosses of Arachis monticola with A. hypogaea Subspecies1.* Peanut Science, 2010. **25**(1): p. 7-12.
5. Koppolu, R., et al., *Genetic relationships among seven sections of genus Arachis studied by using SSR markers.* Bmc Plant Biology, 2010. **10**(1): p. 1-12.
6. Temsch, E.M. and J. Greilhuber, *Genome size variation in Arachis hypogaea and A. monticola re-evaluated.* Genome, 2000. **43**(3): p. 449-451.
7. Bertioli, D.J., et al., *The genome sequences of Arachis duranensis and Arachis ipaensis, the diploid ancestors of cultivated peanut.* Nature Genetics, 2016. **48**(4): p. 438.
8. Maccallum, I., et al., *ALLPATHS 2: small genomes assembled accurately and with high continuity from short paired reads.* Genome Biology, 2009. **10**(10): p. 1-10.
9. Koren, S., et al., *Canu: scalable and accurate long-read assembly via adaptive k-mer weighting and repeat separation.* Genome Research, 2017. **27**(5): p. 722.
10. Salmela, L. and E. Rivals, *LoRDEC: accurate and efficient long read error correction.* Bioinformatics, 2014. **30**(24): p. 3506-3514.
11. Chin, C., et al., *Phased diploid genome assembly with single-molecule real-time sequencing.* Nature Methods, 2016. **13**(12): p. 1050-1054.
12. *https://github.com/ruanjue/wtdbg*.
13. Simão, F.A., et al., *BUSCO: assessing genome assembly and annotation completeness with single-copy orthologs.* Bioinformatics, 2015. **31**(19): p. 3210.
14. Parra, G., K. Bradnam, and I. Korf, *CEGMA: a pipeline to accurately annotate core genes in eukaryotic genomes.* Bioinformatics, 2007. **23**(9): p. 1061-1067.
15. Walker, B.J., et al., *Pilon: An Integrated Tool for Comprehensive Microbial Variant Detection and Genome Assembly Improvement.* Plos One, 2014. **9**(11): p. e112963.
16. Chakraborty, M., et al., *Contiguous and accurate de novo assembly of metazoan genomes with modest long read coverage.* Nucleic Acids Research, 2016. **44**(19): p. 029306.
17. Lam, E.T., et al., *Genome mapping on nanochannel arrays for structural variation analysis and sequence assembly.* Nature Biotechnology, 2012. **30**(8): p. 771-776.
18. *https://bionanogenomics.com/support/software-downloads/.*
19. Li, H. and R. Durbin, *Fast and accurate short read alignment with Burrows-Wheeler transform.* Bioinformatics, 2009. **25**(14): p. 1754-60.
20. Boetzer, M., et al., *Scaffolding pre-assembled contigs using SSPACE.* Bioinformatics, 2011. **27**(4): p. 578-9.
21. Raina, S.N. and Y. Mukai, *Genomic in situ hybridization in Arachis (Fabaceae) identifies the diploid wild progenitors of cultivated (A. hypogaea) and related wild (A. monticola) peanut species.* Plant Systematics & Evolution, 1999. **214**(1-4): p. 251-262.
22. English, A.C., et al., *Mind the Gap: Upgrading Genomes with Pacific Biosciences RS*

Long-Read Sequencing Technology. PLOS ONE, 2012. **7**(11).

23. Luo, R., et al., *SOAPdenovo2: an empirically improved memory-efficient short-read de novo assembler.* Gigascience, 2012. **1**(1): p. 18.
24. Burton, J.N., et al., *Chromosome-scale scaffolding of de novo genome assemblies based on chromatin interactions.* Nature Biotechnology, 2013. **31**(12): p. 1119.
25. Bickhart, D.M., et al., *Single-molecule sequencing and chromatin conformation capture enable de novo reference assembly of the domestic goat genome.* Nature Genetics, 2017. **49**(4): p. 643.
26. Zhang, L., et al., *The Tartary Buckwheat Genome Provides Insights into Rutin Biosynthesis and Abiotic Stress Tolerance.* Mol Plant, 2017. **10**(9): p. 1224-1237.
27. Avni, R., et al., *Wild emmer genome architecture and diversity elucidate wheat evolution and domestication.* Science, 2017. **357**(6346): p. 93-97.
28. Mascher, M., et al., *A chromosome conformation capture ordered sequence of the barley genome.* Nature, 2017. **544**(7651): p. 427.
29. Martin, M., *Cutadapt removes adapter sequences from high-throughput sequencing reads.* Embnet Journal, 2011. **17**(1).
30. Servant, N., et al., *HiC-Pro: an optimized and flexible pipeline for Hi-C data processing.* Genome Biology, 2015. **16**(1): p. 259.
31. Kurtz, S., et al., *Versatile and open software for comparing large genomes.* Genome Biology, 2004. **5**(2): p. R12.
32. Haas, B.J., et al., *De novo transcript sequence reconstruction from RNA-Seq: reference generation and analysis with Trinity.* Nature Protocols, 2013. **8**(8): p. 1494.
33. Chaisson, M.J. and G. Tesler, *Mapping single molecule sequencing reads using basic local alignment with successive refinement (BLASR): application and theory.* BMC Bioinformatics, 2012. **13**(1): p. 238.
34. Grabiele, M., et al., *Genetic and geographic origin of domesticated peanut as evidenced by 5S rDNA and chloroplast DNA sequences.* Plant Systematics & Evolution, 2012. **298**(6): p. 1151-1165.
35. Stalker, H.T., et al., *Variation of isozyme patterns among Arachis species.* Tag.theoretical & Applied Genetics.theoretische Und Angewandte Genetik, 1994. **87**(6): p. 746.
36. Moretzsohn, M.C., et al., *A study of the relationships of cultivated peanut (Arachis hypogaea) and its most closely related wild species using intron sequences and microsatellite markers.* Annals of Botany, 2013. **111**(1): p. 113.
37. Bertioli, D.J., et al., *The Use of SNP Markers for Linkage Mapping in Diploid and Tetraploid Peanuts.* G3 Genesgenetics, 2014. **4**(1): p. 89-96.
38. Shifeng Cheng, et al., 10KP: A Phylodiverse Genome Sequencing Plan, *GigaScience*, 2018, giy013, https://doi.org/10.1093/gigascience/giy013

Tables

Table1. Statistics of pseudochromosomes of *A. monticola*.

	Chr	Length (bp)	No. of gap	Gap length (bp)	Gaps ratio (%)	Anchored percent (%)
	A.mon-A01	118,283,061	1,961	12,923,146	10.93	4.51
	A.mon-A02	84,409,872	1,598	13,652,890	16.17	3.22
	A.mon-A03	123,011,103	2,089	18,448,429	15.00	4.69
	A.mon-A04	106,244,467	2,020	15,031,534	14.15	4.05
	A.mon-A05	123,320,146	1,950	15,552,662	12.61	4.70
A.mon-A	A.mon-A06	98,474,784	1,770	11,764,791	11.95	3.75
	A.mon-A07	72,108,480	1,299	7,250,302	10.05	2.75
	A.mon-A08	39,681,652	442	1,898,702	4.78	1.51
	A.mon-A09	107,717,523	1,889	11,324,084	10.51	4.11
	A.mon-A10	100,634,791	1,847	13,895,555	13.81	3.84
	Un-chr	61,870,352	422	7,811,614	12.63	2.36
	A.mon-B01	140,073,190	2,773	17,354,378	12.39	5.34
	A.mon-B02	124,915,013	2,271	14,941,271	11.96	4.76
	A.mon-B03	160,549,902	2,512	18,727,668	11.66	6.12
	A.mon-B04	147,957,427	2,521	16,939,677	11.45	5.64
	A.mon-B05	121,568,645	2,396	14,347,666	11.80	4.63
A.mon-B	A.mon-B06	154,488,041	2,644	22,222,939	14.38	5.89
	A.mon-B07	136,067,974	2,462	15,804,193	11.61	5.19
	A.mon-B08	138,850,997	2,492	17,429,178	12.55	5.29
	A.mon-B09	163,848,611	2,991	16,573,361	10.12	6.24
	A.mon-B10	147,468,805	2,693	18,369,757	12.46	5.62
	Un-chr	49,370,401	428	7,142,698	14.47	1.88
Unknown	--	103,005,886	972	16,282,706	15.81	--
	Total	2,623,921,123	46,879	325,689,201	12.41	--

Table2. Comparison of assembly results between *A. monticola* and its progenitors.

	A.mon-A	*A.mon*-B	*A. duranensis*	*A. ipaensis*
Genome size (bp)	1,035,756,231	1,485,159,006	1,068,326,401	1,257,035,815
Contig number	18,620	27,431	135,613	123,165
Max length (bp)	1,481,449	1,683,058	221,145	250,973
Min length (bp)	14,852	10,392	10,007	10,021
Contig N50 (bp)	107,702	110,501	22,900	22,562
Contig N90 (bp)	29,116	29,291	3,342	5,216
Gap number	18,005	26,847	134,110	122,617
Gap ratio (%)	12.50	12.11	11.95	7.32
GC content (%)	35.79	36.18	35.81	36.85

Note: only sequences whose length is more than 10 Kb are considered.

Figure Legends

Figure 1. Morphological characters of the *Arachis monticola*. Mature plants in field (A), flowers (B), and pods (C) are shown.

Figure 2. Work flow of assembly of alloteraploid wild peanut (*A. monticola*). We first corrected SMRT subreads by error correction module of Canu based on 36.10 x Pacbio subreads. For subreads aborted by Canu, we corrected them with LoRDEC based on ~50 fold coverage of Illumina short reads. Then we assembled these high quality data using Canu, Falcon and WTDGB, respectively, and used Pilon to polish them. To integrate advantages of different algorithm, we merged the assemblies by Quickmerge. We also curated "chimeric error" of genome assembly combing Pacbio molecules, BioNano data and HiC links, and scaffolded the contigs using SSPACE and IrysView. Further analysis of scaffolds order and orientation through HiC-pro and LACHESIS led to chromosome-length scaffolds. SMRT subreads and short reads were used for gap filling and genome polishing through Pbjelly, GapCloser and Pilon packages. The subgenomes of AA- and BB- genotypes were simply distinguished by the overall macro-synteny between genome assemblies and its corresponding ancestors.

Figure 3. Interaction frequency distribution of Hi-C links among chromosomes. (A) Genome-wide Hi-C map of *A. monticola*. (B) Genome-wide Hi-C map of *A.ipaensis* and *A.duranensis*. We scanned the genome by 500 Kb non-overlapping window as a bin and calculated valid interaction links of Hi-C data between any pair of bins. The log2 of link number was calculated. The distribution of links among chromosomes was exhibited by heatmap based on HiCplotter. The color key of heatmap ranging from light yellow to dark red indicated the frequency of Hi-C interaction links from low to high (0~10).

Table1. Statistics of pseudochromosomes of *A. monticola*.

A. monticola	Chr	Length (bp)	No. of gap	Gap length (bp)	Gaps ratio (%)	Anchored percent (%)
	Amon01	112,346,344	1,450	11,158,127	9.93	4.21
	Amon02	77,452,868	1,177	11,759,290	15.18	2.90
	Amon03	163,787,642	2,087	19,806,873	12.09	6.14
	Amon04	99,310,551	1,496	13,014,239	13.10	3.72
	Amon05	118,952,800	1,506	13,218,499	11.11	4.46
A.mon-A	Amon06	112,343,459	1,581	11,174,828	9.95	4.21
	Amon07	74,464,882	1,007	7,116,888	9.56	2.79
	Amon08	20,755,833	168	414,219	2.00	0.78
	Amon09	105,291,949	1,449	11,482,385	10.91	3.95
	Amon10	82,881,032	1,275	11,966,790	14.44	3.11
	Un-chr	94,127,220	1,347	12,642,337	13.43	3.53
	Amon11	134,981,886	2,047	15,011,108	11.12	5.06
	Amon12	115,993,871	1,658	13,635,813	11.76	4.35
	Amon13	118,646,716	1,524	14,653,267	12.35	4.45
	Amon14	136,096,094	1,829	13,173,787	9.68	5.10
	Amon15	107,897,822	1,734	12,146,235	11.26	4.05
A.mon-B	Amon16	131,809,947	1,807	17,580,447	13.34	4.94
	Amon17	133,410,505	1,863	14,750,241	11.06	5.00
	Amon18	135,971,108	1,903	15,559,945	11.44	5.10
	Amon19	165,135,086	2,391	15,486,856	9.38	6.19
	Amon20	154,326,195	2,130	16,745,212	10.85	5.79
	Un-chr	119,134,814	1,841	18,215,623	15.29	4.47
Unknown	--	151,596,038	2,145	20,207,684	13.33	--
	Total	2,666,714,662	37,415	310,920,693	11.66	--

Table2. Comparison of assembly results between *A. monticola* and its progenitors.

	A.mon-A	*A.mon*-B	*A. duranensis*	*A. ipaensis*
Genome size (bp)	1,061,714,580	1,453,404,044	1,068,326,401	1,257,035,815
Contig number	15,604	21,906	135,613	123,165
Max length (bp)	1,687,026	1,513,691	221,145	250,973
Min length (bp)	17,426	13,964	10,007	10,021
Contig N50 (bp)	127,369	129,638	22,900	22,562
Contig N90 (bp)	34,374	35,007	3,342	5,216
Gap number	140,377	20,727	134,110	122,617
Gap ratio (%)	11.67	11.95	11.95	7.32
GC content (%)	35.87	36.32	35.81	36.85

Note: only sequences whose length is more than 10 Kb are considered.

Click here to download Figure Figure 1.jpg

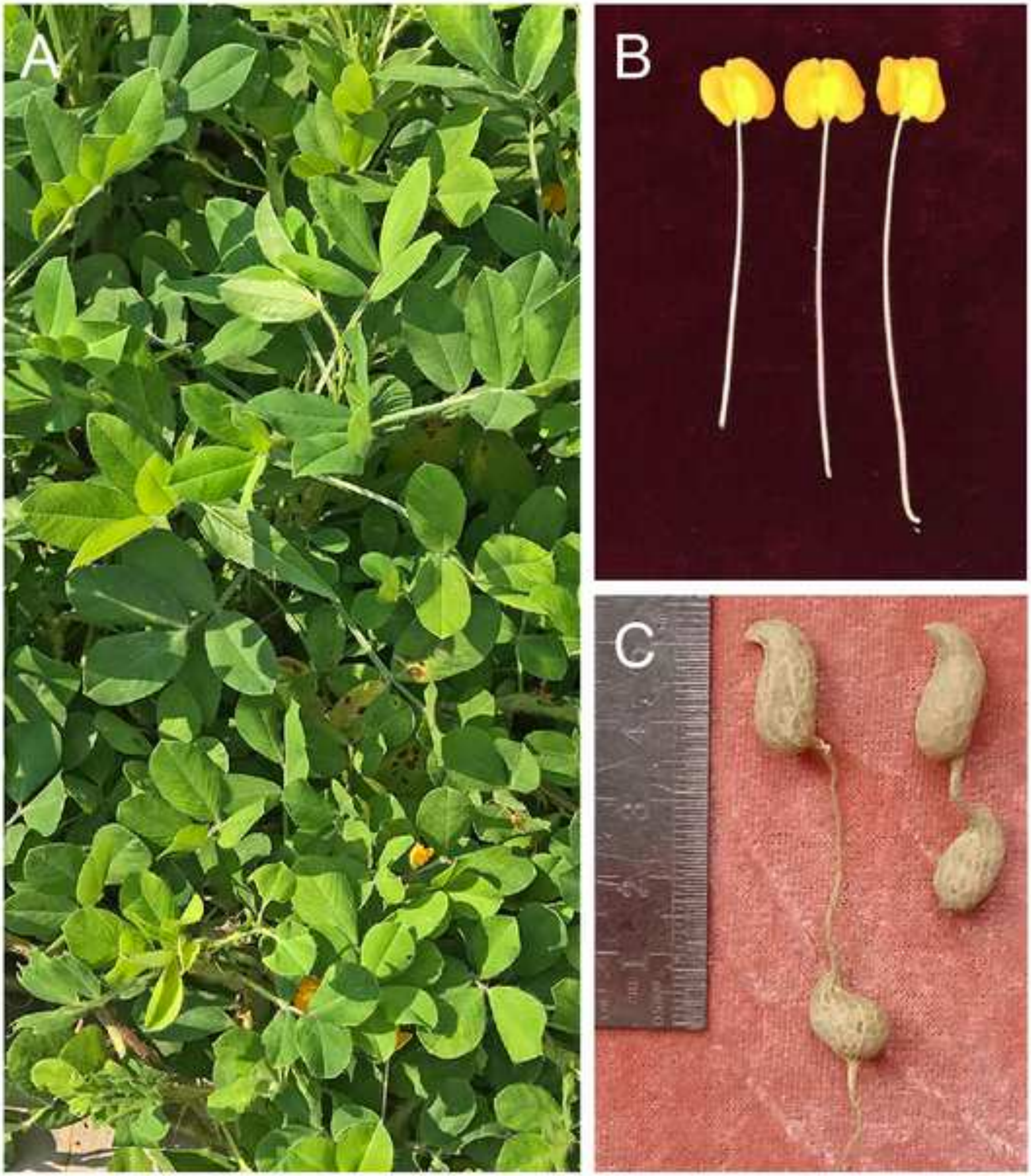

Figure 2. Work flow of assembly of alloteraploid wild peanut.

Click here to download Figure Figure 2..pdf

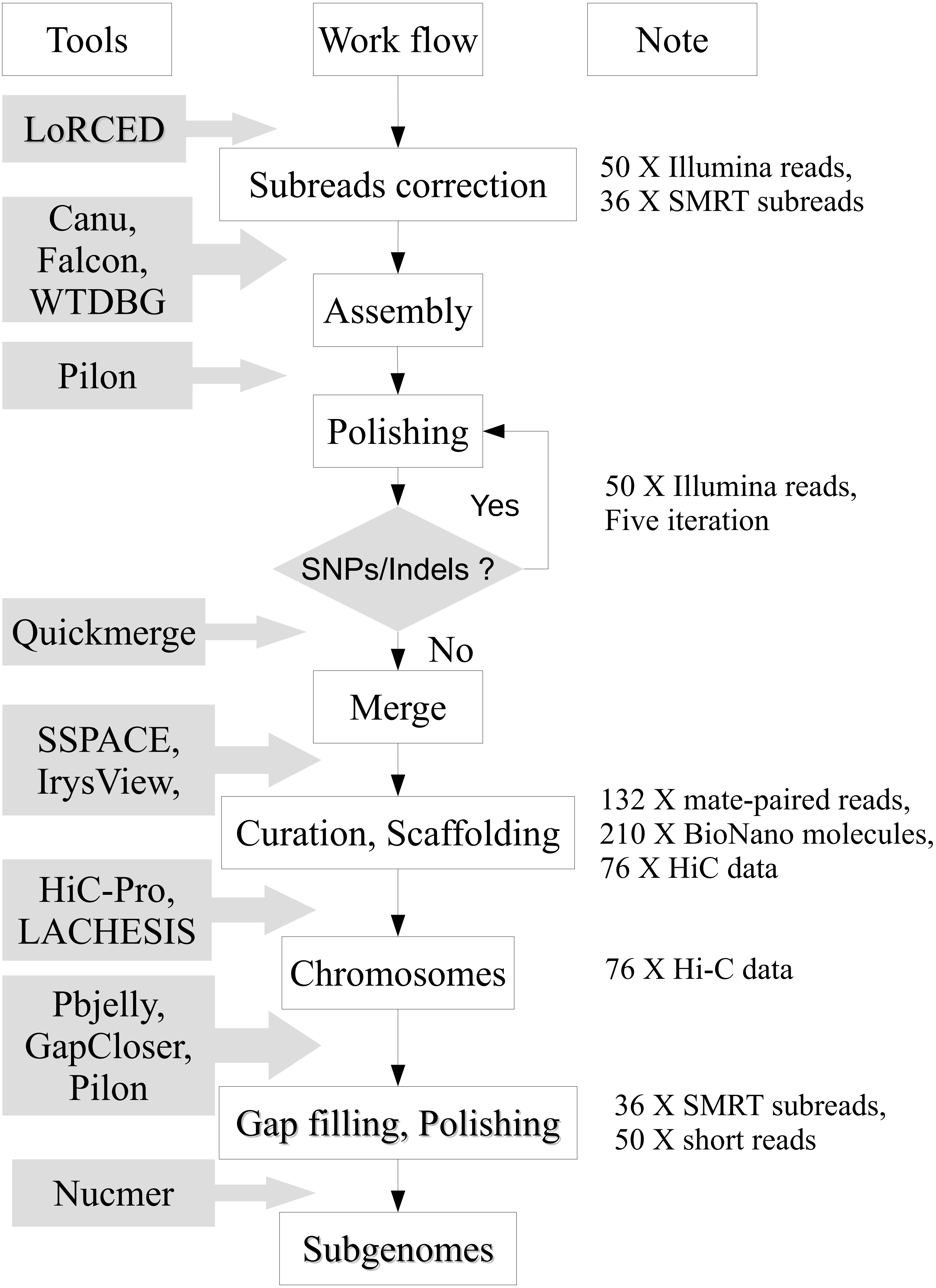

Figure 3A. Genome-wide Hi-C map of A. monticola

Click here to download Figure Figure 3A. Genome-wide Hi-C map of A. monticola.pdf

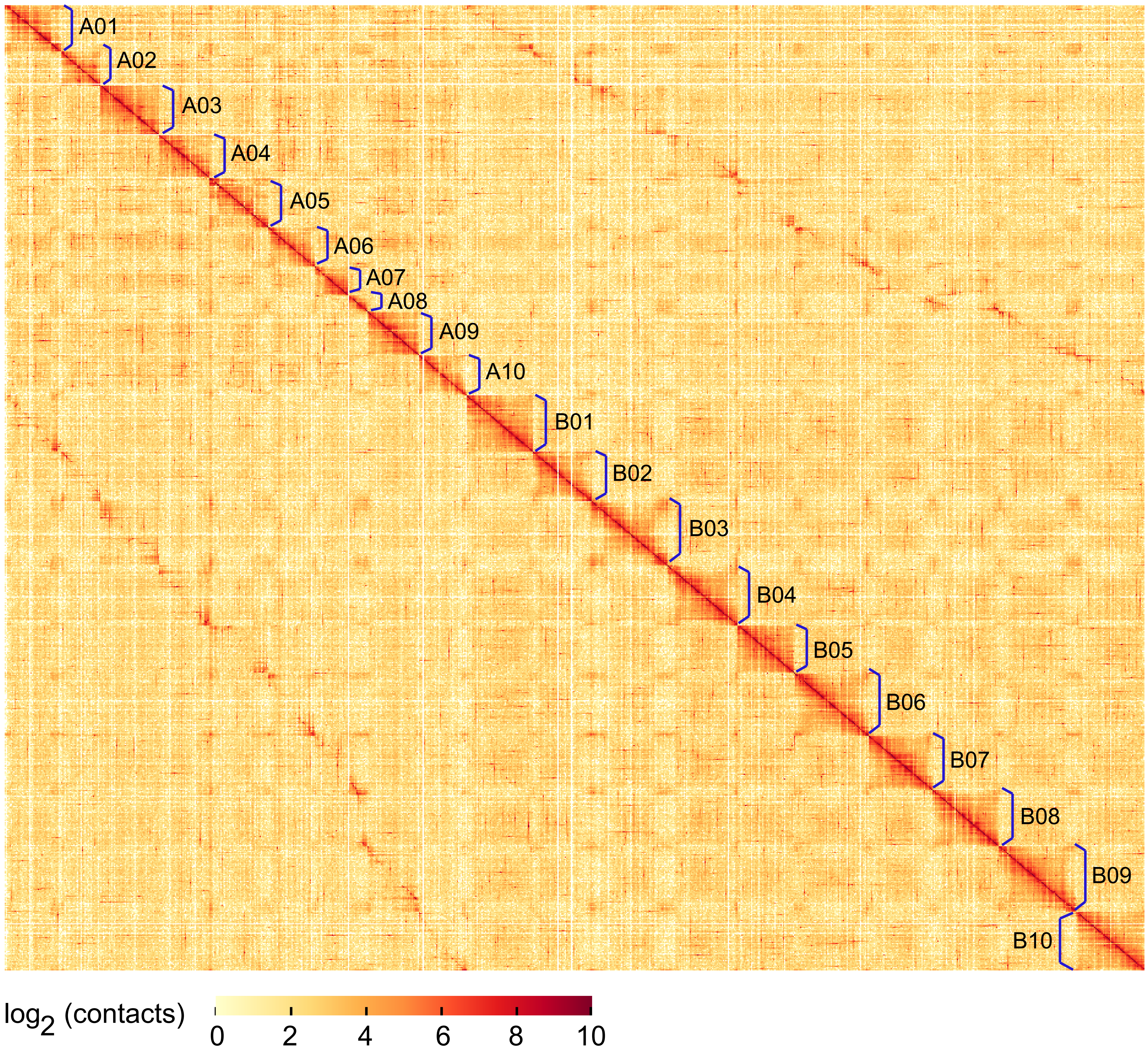

 Click here to download Figure Figure 3B. Genome-wide Hi-C map of A.ip and A.du..pdf

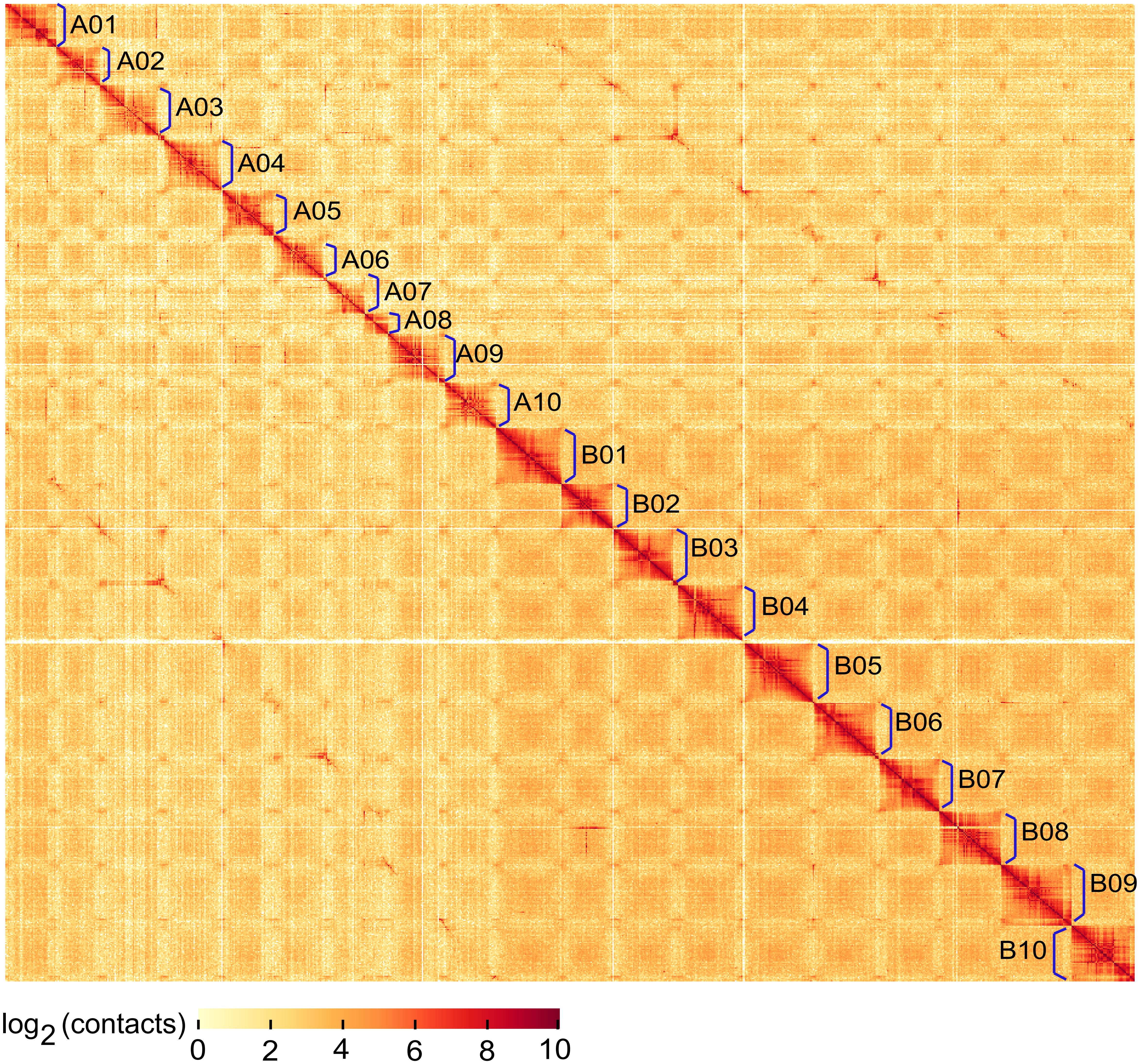

Click here to access/download
Supplementary Material
Supplementary tables v1.22 20180306.xls

Sup figure 1

Click here to access/download
Supplementary Material
Sup figure 1.jpg

Sup figure 2

Click here to access/download

Supplementary Material

Sup figure 2.jpg

Dear Scott,

Thank you for your letter and the review's comments.

We have made correction which we hope it will meet with your approval.

Here are our response to the comments.

In particular please ensure all the supporting data is available and live with sufficient metadata. To ensure reproducibility please ensure all identifiers and accessions are included: for example NCBI taxon IDs, voucher numbers and information on the sample sequenced, ORCID IDs for authors that have them, RRIDs for software resources used (see ftp://penguin.genomics.cn/pub/10.5524/RRID/RRIDlist.pdf), and accessions for any datasets discussed. One of the reviewers has raised issues on the advance of this paper. Data Note articles do not require analysis, but do need sufficient rationale and context, as well as validation and benchmarking.

Response: we have provided the correct accessions in NCBI and GigaDB.

In the introduction please state what motivated you to produced this data, and give some background on the area of research to relate the work to previous studies and help understand its utility and potential for reuse. Where does this dataset fit in current sequenced plant (legume/peanut) species. For validation you will then need sufficient comparisons with these relevant datasets (metrics and some phylogenies). At the end of the paper you need to provide instructions and suggestions of downstream applications that may help other researchers with reuse of the data. This section can also promote discussion on possible ways the data presented might be used in or have a relationship with other areas of research that may not be directly apparent in the work. As a lot of work is being carried out to resolve the phylogeny of plants (see https://doi.org/10.1093/gigascience/giy013) and legumes in particular does this work help with that?

Response: We appreciate your valuable suggestion. The high quality reference genome of allotetraploid peanut is important for evolution,

origin and domestication research of wild and cultivated peanuts, and favorable to peanut breeding, which is helpful for peanut research community. So we added some background in the introduction(Line 62-73, 82-91). The comparisons between our genome datasets and the two diploid wild ancestors had also been done(Sup Figure1-2). At the end of the paper we provided some instructions of the future applications and added the ref.(https://doi.org/10.1093/gigascience/giy013) which is helpful for the phylogeny of plants(Line 378-384).

It is important to stress again that while Data Note articles do not require analysis, they do need sufficient rationale and context, as well as validation and benchmarking.

Relating to this the follow still needs addressing:

a) We have strict policies that all supporting data and intermediate data/results and code enabling reproduction are available. In your draft you state: "All other data are available from the corresponding author at reasonable request". This goes against our policies, so please ensure all these resources are in our FTP server and remove this statement.

Response: We removed this statement and all the data are in the FTP.

b) Regarding the rationale, you have added five sentences in the introduction and two at the end of the paper. For the introduction you need some more context and detail on what motivated you to produce this data, and where it sits in the field so far. The field has been working up to sequence the cultivated peanut, but this has been challenge, and there have been additional studies sequencing the wild varieties (e.g. http://www.pnas.org/content/113/24/6785.long). These few lines do not get that across sufficiently and make it clear that this multi-platform approach is a significant effort in overcoming these challenges.

Response: We added some background in the introduction(Line 62-73, 82-91).

For the final section, does this data help the genome of the cultivated peanut, and as a lot of work is being carried out to resolve the phylogeny of plants (see https://doi.org/10.1093/gigascience/giy013) and legumes in particular does this work help with that?

Response：At the end of the paper we provided some instructions of the future applications and added the ref.38 (https://doi.org/10.1093/gigascience/giy013) which is helpful for the phylogeny of plants(Line 378-384).

c) Regarding the validation, we require sufficient comparisons with these relevant previously published datasets so on top of quality metrics please add some phylogenies. This will really help provide context on the quality and importance of this new data.

Response：The comparisons between our genome datasets and the two diploid wild ancestors had also been done(Sup Figure1-2).

d) Please check a final time for typos. e.g. where you have added the few lines in the introduction there is a random "Section" added at p3, line 65.

Response: The word "Section" is accurate.

Here are our responses to reviewers' comments.

Reviewer #1: This paper reports what appears to be a high-quality genome assembly of the wild tetraploid peanut relative, Arachis monticola. The paper primarily reports the assembly methods and statistics. As far as I am able to tell, the assembly appears to have been made using state-of-the-art methods.

The main weakness (and a very large one) is that the assembly is not accessible. I assume it will be made available after embargo, but as this paper primarily describes the assembly resource, I don't feel that I can adequately assess the resource if it is not available. It appears that the accession IDs provided for GigaDB and GenBank are placeholders. I believe the standard practice is instead to provide actual identifiers provided by those databases - which may be held pending embargo release, depending on release conditions.

Response: we have provided the correct accessions in NCBI and GigaDB including project accession, biosample accession, SRA accessions of Illumina, pacbio and HiC data, and genome sequences accession in Availability of supporting data.

It would also have been nice for the authors to have provided some scientific analysis of the assembly, beyond a simple description of the resource. For example, no report is provided regarding sequence interactions (exchanges) between the subgenomes, or evidence about rearrangements in the progenitor species - or sequence similarity with the diploid progenitors.

Response: We appreciate your valuable suggestion. In this study, we released the genome of an allotetraploid wild peanut firstly for publication in Gigascience as "DATA NOTE" article, and we provided here the circus plot to demonstrate the high consistency of A and B subgenomes in tetraploid peanut with diploid ancestors (*A. ipaensis* and *A. duranensis*) (Sup Figure1-2). More detailed analysis are already under way and an interesting story will show in the following paper.

Specific comments:
Introduction
60: "has a key role on food security within the global society" -- better as "has a key role in food security globally" (on -> in and global society -> globally)

62: "The genus Arachis is unique of subterranean fruit originated" --> The genus Arachis is unique for its subterranean fruit, which originates from

82: "billion basesand" --> : billion bases and

84: occupying --> spanning

Response: we have revised the MS following the advises in line 61, 62, 99 and 101.

Availability of supporting data:
354: At http://gigadb.org, I find no record for RRID:SCR00000 or for SCR00000. This seems to be a placeholder. The correct accession will be needed.

Response: we have revised the MS following the advises in line 388 (Availability of supporting data).

355: "Raw data were deposited in the Sequence Read Archive (SRA00000) for the Arachis monticola genome." -- the accession SRA00000 seems to be a placeholder. At GenBank, I see biosample SAMN05572396 and bioproject PRJNA248910 for Arachis monticola, but from a different research group. The correct accession(s) will be needed.

Response: we have provided the correct accessions in NCBI and GigaDB including project accession, biosample accession, SRA accessions of Illumina, pacbio and HiC data, and genome sequences accession in Availability of supporting data.

Reviewer #2: The genome assembly for Arachis monticola is very important for Arachis species research and therefore peanut research will be highly benefited by this resource. I have following comments for the authors:
1. Needs proper justification that why A. monticola is so important for peanut improvement as this wild relative of cultivated peanut

Response: We agree with this comment and associated content has been added in the revised manuscript (line 62~73, 82~91).

2. Details on the genotype/accession not provided throughout MS not provided

Response: We have added the genotype/accession of the sequencing samples in MS, from line 105 to 107.

3. The information provided in the first para of Results section was again repeated in detail in rest of the sections of the Results...looked much text repetitive

Response: We have rewrote the Results and removed the redundant contents from line 90~102 of previous version to line 113~117 of revised version.

4. The major problem with this MS is that this MS appears totally descriptive in nature and no scientific advancement been provided to the available understanding/knowledge

Response: We have revised the MS as review's advises from line 62~73, 82~91 in the revised version.

5. Sequencing genome is not just enough for publications..until unless it makes some sense for the research community

Response: We follow the review's points. Although previous publication of reference genome sequences of peanut diploid ancestors (A. ipaensis and A. duranensis) provide the valuable understanding and knowledge of peanut/legume and facilitated the peanut research, all the cultivated peanut varieties are allotetraploids. The high quality reference genome of allotetraploid peanut is important for evolution, origin and domestication research of wild and cultivated peanuts, and favorable to peanut breeding, which is helpful for peanut research community. So we published the genome assembly as data note as soon as possible, which would benefit to community and scientists. The further research including subgenomes evolution, origin and domestication also have been planned. Here, we provide the circus plot to demonstrate the high consistency of A and B subgenomes in tetraploid peanut with diploid ancestors (*A. ipaensis and A. duranensis*).

6. The MS in present form does not add value to much for peanut research community except providing genome assembly of A. monticola for research

Response: Please refer to the response to comment 5.

7. Authors are suggested to include some evolutionary, origin and domestication related analysis and results for improving this MS

Response: Please refer to the response to comment 5.

I greatly appreciate your help and the referees concerning improvement to this paper. I hope the revised manuscript is now suitable for publication. I'm looking forward to hearing from you soon.

Sincerely yours,

Dongmei,Yin
