# Supplementary material for: Genome of an allotetraploid wild peanut Arachis monticola: a de novo assembly
Source: Gigascience. 2018 Jun 19;7(6):giy066. doi: 10.1093/gigascience/giy066 (PMC6009596; doi:10.1093/gigascience/giy066)
Supplement: GIGA-D-18-00025_Revision_3.pdf [file giy066_giga-d-18-00025_revision_3.pdf]

GigaScience

Genome of an allotetraploid wild peanut Arachis monticola: a de novo assembly

--Manuscript Draft--

**Manuscript Number:**	GIGA-D-18-00025R3
**Full Title:**	Genome of an allotetraploid wild peanut Arachis monticola: a de novo assembly
**Article Type:**	Data Note
**Funding Information:**	National Natural Science Foundation of China (31471525) — Dr. Dongmei Yin<br>Key program of NSFC-Henan United Fund (U1704232) — Dr. Dongmei Yin<br>key scientific and technological project in Henan Province (161100111000;S2012-05-G03)) — Dr. Dongmei Yin<br>Innovation Scientists and Technicians Troop Construction Projects of Henan Province (2018JR0001) — Dr. Dongmei Yin
**Abstract:**	Arachis monticola (2n = 4x = 40) is the only allotetraploid wild peanut within section Arachis, with an AABB-type genome of about ~2.7 Gb. The AA-type subgenome is derived from diploid wild peanut Arachis duranensis, and the BB-type subgenome is derived from diploid wild peanut Arachis ipaensis. A. monticola is regarded either as the direct progenitor of the cultivated peanut or as an introgressive derivative between the cultivated peanut and wild species. The large polyploidy genome structure and enormous nearly identical regions of the genome make the assembly of chromosomal pseudomolecules very challenging. Here we report the first reference quality assembly of A. momticola genome, using a series of advanced technologies. The final whole genome of A. monticola is ~2.62 Gb, and has a contig N50 and scaffold N50 of 106.66 Kb and 124.92 Mb, respectively. The vast majority (91.83%) of the assembled sequence was anchored onto the 20 pseudo-chromosomes and 96.07% of assemblies were accurately separated into AA- and BB- subgenomes. We demonstrated efficiency of the current state of the strategy for de novo assembly of the highly complex allotetraploid species, wild peanut (A. monticola), based on whole-genome shotgun sequencing, single molecule real-time (SMRT) sequencing, high-throughput chromosome conformation capture (Hi-C) technology and BioNano optical genome map. These combined technologies produced reference-quality genome of the allotetraploid wild peanut, which is valuable for understanding peanut domestication and evolution within Arachis genus and among legume crops.
**Corresponding Author:**	Dongmei Yin<br><br>CHINA
**Corresponding Author Secondary Information:**	
**Corresponding Author's Institution:**	
**Corresponding Author's Secondary Institution:**	
**First Author:**	Dongmei Yin
**First Author Secondary Information:**	
**Order of Authors:**	Dongmei Yin<br>Changmian Ji<br>Xingli Ma<br>Hang Li<br>Wanke Zhang

Powered by Editorial Manager® and ProduXion Manager® from Aries Systems Corporation

	Song Li
	Fuyan liu
	Kunkun Zhao
	Fapeng Li
	Ke Li
	Longlong Ning
	Jialin He
	Yuejun Wang
	Fei Zhao
	Yilin Xie
	Hongkun Zheng
	Xinguo Zhang
	Yijing Zhang
	Jinsong Zhang
**Order of Authors Secondary Information:**	
**Response to Reviewers:**	Reviewer reports:<br>Reviewer #1: Thanks to the authors for the last round of revisions. The remaining significant issue has been addressed - that is, submission of the assembly to GenBank.<br><br>There is still a very minor point at lines 46-47 - something that I think should be at the author's discretion, but I mention for completeness. My point was not that the estimate of 97.04% was necessarily incorrect, but that the use of four significant digits is unwarranted (it is impossible to know whether 97.04% is correct given the data), because the size estimate for the genome has low precision: "about 2.7 Gb". I just point it out as a suggestion to clean up something that looks unprofessional. See the discussion of significant digits here for example: http://ec.europa.eu/eurostat/statistics-explained/index.php/Tutorial:Rounding_of_numbers<br><br>Response：We really appreciate your kind help for improving our manuscript. We have deleted the setence" representing 97.04% of the estimated genome size" following the advises in line 46-47.
**Additional Information:**	
**Question**	**Response**
Are you submitting this manuscript to a special series or article collection?	No
**Experimental design and statistics**<br><br>Full details of the experimental design and statistical methods used should be given in the Methods section, as detailed in our Minimum Standards Reporting Checklist. Information essential to interpreting the data presented should be made available in the figure legends.<br><br>Have you included all the information requested in your manuscript?	Yes

**Resources**<br><br>A description of all resources used, including antibodies, cell lines, animals and software tools, with enough information to allow them to be uniquely identified, should be included in the Methods section. Authors are strongly encouraged to cite Research Resource Identifiers (RRIDs) for antibodies, model organisms and tools, where possible.<br><br>Have you included the information requested as detailed in our Minimum Standards Reporting Checklist?	Yes
**Availability of data and materials**<br><br>All datasets and code on which the conclusions of the paper rely must be either included in your submission or deposited in publicly available repositories (where available and ethically appropriate), referencing such data using a unique identifier in the references and in the "Availability of Data and Materials" section of your manuscript.<br><br>Have you have met the above requirement as detailed in our Minimum Standards Reporting Checklist?	Yes

Powered by Editorial Manager® and ProduXion Manager® from Aries Systems Corporation

Manuscript

Click here to download Manuscript Genome of an allotetraploid wild peanut_SCEedits.doc

DATA NOTE

Genome of an allotetraploid wild peanut *Arachis monticola*: a de novo assembly

Dongmei Yin [1*†], Changmian Ji [2,†], Xingli Ma[1,†], Hang Li[2], Wanke Zhang[3], Song Li[2], Fuyan Liu[2], Kunkun Zhao[1], Fapeng Li[1], Ke Li[1], Longlong Ning[1], Jialin He[1], Yuejun Wang[4], Fei Zhao[4], Yilin Xie[4], Hongkun Zheng[2], Xingguo Zhang[1], Yijing Zhang[4], Jinsong Zhang[3*]

1 College of Agronomy, Henan Agricultural University, Zhengzhou 450002, China

2 Biomarker Technologies Corporation, Beijing 101300, China

3 State Key Lab of Plant Genomics, Institute of Genetics and Developmental Biology, Chinese Academy of Sciences, Beijing 100101, China

4 National Key Laboratory of Plant Molecular Genetics, CAS Center for Excellence in Molecular Plant Sciences, Institute of Plant Physiology and Ecology, Shanghai Institutes for Biological Sciences, Chinese Academy of Sciences, Shanghai 200032, China

*Corresponding author. Dongmei Yin, Xingguo Zhang, College of Agronomy, Henan Agricultural University, Rd. Wenhua No. 95, Zhengzhou, Henan 450002, P. R. China.Tel: 0086-371-63558122; E-mail: yindm@126.com; Jinsong Zhang, State Key Lab of Plant Genomics, Institute of Genetics and Developmental Biology, Chinese Academy of Sciences, Beijing 100101, P. R. China. Tel: 0086-10-64807601; E-mail: jszhang@gentics.ac.cn;

† Equal contribution

ORCIDs: Dongmei Yin: ORCID: 0000-0002-6200-8388; Changmian Ji: 0000-0001-8781-4063;

Abstract

Arachis monticola (2n = 4x = 40) is the only allotetraploid wild peanut within the *Arachis* genus and section, with an AABB-type genome of about ~2.7 Gb in size. The AA-type subgenome is derived from diploid wild peanut *Arachis duranensis*, and the BB-type subgenome is derived from diploid wild peanut *Arachis ipaensis*. *A. monticola* is regarded either as the direct progenitor of the cultivated peanut or as an introgressive derivative between the cultivated peanut and wild species. The large polyploidy genome structure and enormous nearly identical regions of the genome make the assembly of chromosomal pseudomolecules very challenging. Here we report the first reference quality assembly of the *A. monticola* genome, using a series of advanced technologies. The final whole genome of *A. monticola* is ~2.62 Gb, and has a contig N50 and scaffold N50 of 106.66 Kb and 124.92 Mb, respectively. The vast majority (91.83%) of the assembled sequence was anchored onto the 20 pseudo-chromosomes and 96.07% of assemblies were accurately separated into AA- and BB- subgenomes. We demonstrated efficiency of the current state of the strategy for de novo assembly of the highly complex allotetraploid species, wild peanut (*A. monticola*), based on whole-genome shotgun sequencing, single molecule real-time (SMRT) sequencing, high-throughput chromosome conformation capture (Hi-C) technology and BioNano optical genome maps. These combined technologies produced reference-quality genome of the allotetraploid wild peanut, which is valuable for understanding the peanut domestication and evolution within the *Arachis* genus and among legume crops.

Introduction

Peanut (*Arachis hypogaea* L.) is widely cultivated in subtropical and tropical regions as a plant-based resource for protein and edible oil, which has a key role in global food security. The genus *Arachis* is unique for its subterranean fruit, which originated in South America and has ~80 described species divided into nine sections based on their morphology, cross compatibility relationships and geographical distribution [1]. Section *Arachis* is of particular interest because it contains 30 diploid wild species, one tetraploid wild species (*A. monticola*) and cultivated peanut (*A. hypogaea*) (2n=4x=40). *A. monticola* was distinct from accessions of *A. hypogaea* with high genetic identity [2, 3]. Moreover, hybrids between *A. monticola* and *A. hypogaea* are fertile [4]. *A. monticola* is considered a distinct species from *A. hypogaea* based mainly on its fruit structure, which has an isthmus separating each seed, resembling the diploid wild species [5, 36]. Comparison of the genomes among the *A. monticola*, *A. hypogaea* and wild species should shed light on the evolutionary and/or domesticated events undergoing in the cultivated species.

As a relatively young allotetraploid species, the genome of wild peanut *A. monticola* exhibits complexity with an AABB-type genome of about ~2.7 Gb [6] and shares many regions of high similarity between its two subgenomes [7]. Challenges are present for its genome assembly due to the large polyploid genome structure and highly homologous genomic sequences. Because of these difficulties, sequencing of the diploid ancestors *A.ipaensis* and *A.duranensis* was first completed [7]. The total

assembled genome sizes were 1.025 Gb and 1.338 Gb respectively for the two species, with a N50 contig length of 22 Kb, using paired-end Illumina sequencing. All *A.ipaensis* pseudomolecules were larger than their *A.duranensis* counterparts and *A.ipaensis* may be a direct descendant contributing to the B subgenome of the cultivated peanut [7]. Although previous publications of reference genome sequences of peanut diploid ancestors (*A. ipaensis* and *A. duranensis*) provide valuable insight and knowledge of peanut/legumes and have facilitated peanut research, all the cultivated peanut varieties are allotetraploids. A high quality reference genome of an allotetraploid peanut is important for evolution, origin and domestication research of wild and cultivated peanuts, and a favorable resource for peanut breeding, making it an important target for the entire peanut research community.

In this study, we used a series of advanced technologies, including whole-genome shotgun sequencing, single molecule real-time (SMRT) sequencing, high-throughput chromosome conformation capture (Hi-C) technology and BioNano optical genome mapping, to generate a high quality genome sequence for the tetroploid wild peanut species *A. monticola*. By combining these very long reads with highly accurate short reads, we have been able to produce an assembly of this tetroploid wild species (*A. monticola*) genome. In total, we used 767.25 billion bases and 210.83 fold genome coverage of BioNano data for the genome assembly. Finally, we generated a 2.62 Gb assembly, spanning 97 % of the estimated genome size for *A. monticola*.

Results

Arachis monticola is an allotetraploid wild peanut species and has features different from the tetraploid cultivated peanut (Fig. 1). Line PI 263393 was selected for genome sequencing. The peanut plants were grown in growth chamber with 25°C, and DNA was extracted from fresh leaves of 30 days old wild peanut seedlings. To create the *Arachis monticola* genome assembly, we generated four extremely large primary data sets including 462.87 Gb Illumina reads (Sup table 1a), 11.5 million SMRT long reads as ~91.71 Gb (Sup table 1b), 2.88 million (~596.26 Gb) high quality BioNano optical molecules (Sup table 1c) and 76.54-fold coverage of the genome of Hi-C data (Sup table 1d). All the reads were generated from the same *A.monticola* line. Taking advantage of integrated technologies, we achieved 2.62 Gb high quality reference genome of wild peanut with 20 pseudo-chromosomes (Table 1 and Sup table 2c), and successfully distinguished two subgenomes (*A. mon-A* and *A.mon-B*) , corresponding to its diploid progenitors *A.ipaensis* and *A.duranensis*, respectively (Sup table 2d).

Initial genome assembly

An independent WGS (whole genome sequencing) assembly was executed using Allpath-LG v1.4 (Allpath-LG, RRID:SCR_010742) [8] to increase the lengths of scaffolds and to fill gaps in the *A. monticola* assembly. Eleven paired-end and mate-paired libraries ranging from 200 bp to 17 Kb were constructed and sequenced (Sup table 1a). From 171 fold coverage reads (~462.87 Gb), we assembled into 1.66 Gb results with scaffold N50 and contig N50 of 369.06 Kb and 16.17 Kb, respectively

(Sup table 2a).

We also assembled the *A. monticola* genome using 97.71 Gb long Pacific Biosciences (PacBio) reads, covering approximately 36.10 fold coverage of genome size (Sup table 1b). Because of a high error rate of PacBio reads, we first corrected these by error correction module of Canu v1.5 [9] based on 36.10 x Pacbio subreads. For subreads aborted by Canu, we corrected them with LoRDEC v0.5 [10] based on ~50 fold coverage of Illumina short reads. Finally, we retained 34.07 fold coverage of high quality subreads (92.78 Gb) and independently assembled them with Falcon v0.7 [11], WTDBG v1.2.8 [12] and Canu v1.5 [9]. The assembled size from Falcon, WTGDB and Canu are 1.88 Gb, 1.96 Gb and 2.26 Gb, respectively. The contig N50 of assembly results was 81.5 Kb, 82.8 Kb and 109.2 Kb, respectively for the three methods (Sup table 2b). The completeness assessment of these assembles through Benchmarking Universal Single-Copy Orthologs (BUSCO) databases (BUSCO, RRID:SCR_015008) [13] and Core Eukaryotic Genes Mapping Approach (CEGMA， RRID:SCR_015055) [14] showed that more than 96% CEGs and 90% of complete BUSCOs are detectable, suggesting the high completeness of the assembly results. We then polished the consensus sequence of three assemblies based on 50 x Illumina pair-end reads using Pilon v1.22 software [15]. To take advantage of assemblies from different tools and generate more contiguity and connectivity results, we merged them together with quickmerge v0.2 package [16]. The strict conditions were considered in this step to avoid chimeric errors. We obtained a genome of 2.24 Gb with contig N50 and longest contig of 120.61 Kb and 1.89 Mb, respectively (Sup table 2b).

Physical map construction

To develop a robust physical map for the allotetraploid wild peanut that could be helpful to place sequence contigs on chromosomes and to determine the physical length of gaps between them [17], we constructed BioNano optical genome map libraries for the sequencing genotype from the fresh leaves. From the enzyme density and distribution assessment of genome sequences using Label Density Calculator v1.3.0 (BioNano Genomics, CA, US), we adopted the Nt.BspQI nickase for optical map library construction. The basic process of BioNano raw data was conducted using the IrysView v2.5.1 package (BioNano Genomics, CA, US). Molecules whose lengths are more than 150 Kb (with label SNR >= 3.0 and average molecule intensity < 0.6) were retained for further genome assembling. We obtained 2.8 million (~596.26 Gb) high quality optical molecules, accounting for ~210 x coverage of genome size (Sup table 1c). The N50 of the molecules is 210.83 Kb (Sup table 1c). On the basis of the label positions on single DNA molecules, de novo assembly was performed by a pairwise comparison of all single molecules and overlap-layout-consensus path building, which adopted by IrysView v2.5.1 assembler [18]. The parameter set for large genomes was used for assembly with the IrysView software. We considered only molecules containing more than seven nicking enzyme sites for assembly (min label per molecule: 8). A *p* value threshold of 1e-8 was used during the pairwise assembly, and 1e-9 for extension and refinement steps and 1e-12 for merging contigs were adopted. The resulting physical map covers approximately 2.65 Gb (around 98.15%

of the 2.7 Gb genome size). We generated 1,404 optical map-based scaffolds with N50 of 3.4 Mb for *A. monticola* (Sup table 1c). The high quality optical map would be used for genome curation and hybrid assembly with SMRT-based assembly, combing the MP links and Hi-C data.

Scaffold construction and curation

Total of nine mate-pair libraries ranging from 3 Kb to 17 Kb fragments were prepared for scaffolds, which accounted for ~132 fold coverage of previous estimated genome size (2.7 Gb) [6] (Sup table 1a). To decrease chimeras in the initial assembly results, we mapped the different fragment mate-paired data to the contigs using BWA v0.7.10 (BWA, RRID:SCR_010910) [19], considering only unique mapping reads for further scaffolds construction. Further scaffolding was performed by SSPACE v3.0 (SSPACE, RRID:SCR_005056) [20]. Contigs are assembled into scaffolds with mate-pair (MP) information, estimating gaps between the contigs according to the distance of MP links. Two contigs supported by at least 3 reasonable MP links in each fragment libraries (insert size +- 5SD) were joined as a scaffold. We assembled 29,454 contigs into 9,157 scaffolds with large reasonable intra-gaps sequences (Sup table 2c). In this step, we obtained 2.35 Gb assembly results for *A. monticola*, whose scaffold N50 and L50 are 491.06 Kb and 1,396, respectively (Sup table 2c).

As a relatively young allotetraploid species, the genome of *A. monticola* is particularly complicated especially considering the phenomenon of partially homologous sequences between its two subgenomes [7, 21]. The assembly results of

allotetraploid genome from SMRT reads may introduce lots of chimeric errors from high homologous and/or large repeated regions of *A.monticola*. The optical map of single molecules from BioNano Genomics' Irys® System could assemble large homologous and repeated regions, taking advantage of its super long molecule reads. As a result, detection of conflicts between contigs/scaffolds and genome map, and correction of the potential errors are strongly necessary and feasible.

To ascertain the quality of assembly results, we generated an *in silico* map of merged results by Knickers v1.5.5.0 program [18] with Nt.BspQI nickase. From the comparison between the contigs/scaffolds and optical maps by RefAligner v5122 [18], we identified 610 conflicts. Next Generation Mapping (NGM-HS) was used to resolve conflicts between the sequence and optical map assemblies by breaking conflict point of assembly. Conflicts were identified based on chimeric score of a conflict junction, mate-pairs information and SMRT molecules alignment result, which is near the conflict junctions on the optical genome map. The chimeric score of conflict junction is defined as the percentage of BioNano molecules that fully align to the 50 kb flanks of optical map. If the chimeric scores of the conflict junction were ≥ 30, and more than two fully aligned optical molecules located across the conflict junction of genome map, we suggested a candidate chimerical error in scaffold/contig sequence. The alignment results of conflict regions were visualized in IrysView [18] for manual investigation. Knickers, RefAligner, and IrysView were obtained from BioNano Genomics [18]. Further investigation of mate-paired links and SMRT molecules alignment would assist to make a decision of cutting on selected sequences. If the

mate-pair relationship (3Kb~17Kb) of 10 Kb flanks of conflict junction is in disagreement, or less than 5 coverage of fully aligned Pacbio molecules are across this region, we suggested breaking the point. We considered the consistent soft-clip sites of SMRT molecules on reference sequence as accurate break point. All proposed cuts were manually evaluated using BioNano molecule-to-genome map alignments, SMRT molecule-to-sequence contig alignments, and mate-paired libraries mapping results based on integrated graphic platform. Of these conflicts, 600 were chimeric in the long reads assembly, and 10 were left unresolved. After chimeric correction, we assembled the 6,262 hybrid scaffolds based on genome map hybrid assembly. The genome size of *A. monticola* is 2.62 Gb, with scaffold N50 of 1.51 Mb (Sup table 2c).

Gap filling and SMRT-error correction

To improve the contiguity of assembly results, we fulfilled the gap filling process combined SMRT sequencing data, Illumina data. PBJelly [22] was used to fill gaps using approximately 34.07 fold coverage of error- corrected SMRT sequencing data from initial genome assembly step. Then we further filled retaining gaps using 39 fold coverage pair-end data (Sup table 1a), along with *de Bruijn* graph analysis to detect instances where a unique path of reads spanned a gap, implemented with Gapcloser v1.12 of SOAPDenovo packages (GapCloser, RRID:SCR_015026) [23]. During the gap-filling procedure, 42.87 Mb gaps were filled by SMRT long reads and Illumina data.

To ensure base-pairing accuracy of assembly results from SMRT molecules, we further polished the consensus sequence after the construction of the pseudomolecules based on ~105 Gb Illumina pair-end reads using Pilon [15]. A total of 5,607 kb bases, including SNPs and small Indels, were corrected, of which 0.21% were small indels.

Pseudomolecules construction and sub-genome identification

High-throughput chromosome conformation capture (Hi-C) technology enables the generation of genome-wide 3D proximity maps and is an efficient and low-cost strategy for sequences cluster, ordered and orientation for pseudomolecule construction [24]. This technology has been successfully applied in recent complex genome projects including goat [25], Tartary buckwheat [26], wild emmer [27], and barely [28]. We constructed three Hi-C fragment libraries ranging from 300-700 bp and sequenced them using the Illumina X-TEN platform (Illumina, San Diego, USA) for pseudomolecules construction. Mapping of Hi-C reads and assignment to restriction fragments were performed as described elsewhere [24]. Briefly, adapter sequences of raw reads were trimmed with cutadapt v1.0 (cutadapt, RRID:SCR_011841) [29] and low quality PE reads were removed for clean data. The clean Hi-C reads, accounting for ~ 60 fold coverage of *A. monticola* genome, were mapped to the assembly results with bwa align v0.7.10 (BWA, RRID:SCR_010910) [19] (Sup table 1d). Only uniquely aligned pairs read whose map quality is more than 20 were considered for further analysis. Duplicate removal, sorting and quality assessment were performed with HiC-Pro v2.8.1 [30]. The 21.98 % of Hi-C data was

valid interaction pairs. Raw counts of Hi-C links were aggregated in 50 kb bins and normalized separately for intra- and inter- chromosomal contacts using LACHESIS [24]. We clustered the sequences into initial 20 groups according to threshold of the contact frequency. For each group, we clustered the sequences in 5 subgroups and independently decided the order and orientation of sequences based on contact probability of each sub-groups. The whole order and orientation subgroup was considered as super-bin and recalculated for the interaction matrices for each group. Then LACHESIS [24] was used to assign the order and orientation of each group. Based on 76.54 fold coverage of Hi-C data, the vast majority (91.83%) of the assembled sequence was anchored onto the 20 pseudo-chromosomes by frequency distribution of valid interaction pairs (Table 1).

Benefiting from the published genomes of *A. duranensi*s and *A. ipaensis,* the donors of alloteraploid peanut, we are able to directly identify the corresponding subgenomes based on the whole genome comparison between the assembly results of *A. monticola* and the two wild diploid peanuts. We aligned the assembly results to its ancestral genomes with Mummer v2.23 [31] and successfully distinguished more than 96.07% of sequences into *A.mon*-A and *A.mon*-B subgenomes (Table 1). Finally, the subgenome size of *A.mon* -A and *A.mon* -B is 1,035.76 Mb and 1,485.16 Mb, respectively, which is comparable to that of their ancestors *A. duranensi*s and *A. ipaensis* (Table 2; Sup table 2d).

Genome quality assessment

Completeness of gene-space representation was evaluated based on the plants dataset

of the BUSCO database with the BUSCO pipeline v3.0.2 (BUSCO, RRID:SCR_015008) [13]. The results showed that 91.67% of complete gene models could be detected in *A. monticola* genome (Sup table 3a). Comparison analysis suggested that the gene region completeness of assemblies is slightly better than their corresponding progenitors (Sup table 3a).

CEGMA [14] provides a simple method to rapidly assess genome completeness. It comprises a set of highly conserved, single-copy genes, present in all eukaryotes, including 458 core eukaryotic genes (CEGs) and 248 of which are highly conserved CEGs. CEGMA v.2.3 (CEGMA, RRID:SCR_015055) analysis [14] suggested that 96.72% of CEGs could be found in the *A. monticola* assembly results, which is comparable to that of their corresponding ancestor with 98.69% (Sup table 3b).

Besides the normal BUSCO [13] and CEGs [14] estimation, transcriptome data of *A. monticola* can also be used for genome completeness assessment. We assembled the 11.96 Gb pooled transcriptome data from root, stem, leaf, flower and seed of *A. monticola* into unigenes using Trinity v2.1.1 (Trinity, RRID:SCR_013048) [32] (Sup table 3c). We also collected unigenes of *A. hypogaea* which generated from developmental transcriptome map (https://www.peanutbase.org/download). We finally obtained 44,205 unigenes whose lengths are more than 500 bp (Sup table 3d). Of which, 43,961 (99.45%) could be supported by the assembly results.

The completeness of the genome assembly was revealed by sequenced bases from aligned along the entire length of the assembly. We remapped the Illumina short reads, RNAseq data and PacBio subreads to the assembly results of *A. monticola*,

respectively. For Illunima short reads and RNAseq data, we aligned paired-end reads to the genome of *A. monticola* by bwa-mem of BWA v0.7.10 (BWA, RRID:SCR_010910) [19] and found that more than 98.47% and 92.21% of them could be correctly remapped to assembly results, respectively (Sup table 3e). We then remapped the error correction SMRT molecules from genome assembly data to assembly results of *A. monticola* by blasr v5.3 [33] and found that 92.16% of subreads had best alignments in assembly results (Sup table 3e).

To evaluate the genome accuracy, we also randomly selected 20 SMRT molecules longer than 45 Kb and aligned to genome sequence. The coverage and identity of all molecules have greater than 99% and 91%, respectively (Sup table 3f). Additionally, the genome-wide Hi-C heatmap of *A. monticola* which shown by HiCplotter at 500 Kb resolution exhibited as expectation that the frequency of intra-chromosome interactions rapidly decrease with linear distance (Fig. 3A). From the same Hi-C data, similar genome-wide interactions map was observed for its ancestors *A. ipaensis* and *A. duranensis* (Fig. 3B). These comparison analysis suggested the high accuracy of *A. monticola* assemblies.

The assembly results achieved a high level of contiguity and connectivity for SMRT molecules, Illumina data, BioNano-genome map and Hi-C data based on hybrid assembly of allotetraploid wild peanut genome. More than 91.83 % of the assemblies were in ordered orientation in 20 pseudomolecules of two subgenomes, ranging from 39.68 Mb to 163.85 Mb (Table 1; Fig. 3A). The remaining 8.17% of the genome assembly was contained in 3,217 smaller scaffolds of at least 10 Kb.

Discussion

*Arachis monticola(*AABB-type genome, 2n = 4x = 40) is the only allotetraploid wild peanut within section *Arachis*, and is regarded either as the direct progenitor of the cultivated peanut or as an introgressive derivative between the peanut and wild species [34, 35]. It is compatible with cultivated peanut in breeding whereas its wild type structure of fruits supports the maintenance of *A. monticola* as a separate taxonomic species [36, 37]. The generation of whole genome assemblies for *A. monticola* will provide basis for the analysis of these interesting events among the genus *Arachis* during selection and/or domestication.

We sequenced 171.44-fold genome coverage of a wild genotype *A. monticola* from 11 Illumina paired-end (PE) and meta-paired (MP) libraries, ranging from 200 bp to 17 Kb fragments (Sup table 1a). A total of ~462.87 Gb short reads enabled us to assemble 1.996 Gb *A. monticola* genome (Sup table 2a). We also generated a 36 fold sequencing coverage of *A. monticola* genome using 30 SMRT cells on the PacBio RS II and Sequel platforms (Sup table 1b). Production of 11.5 million very long reads allowed us to generate a genome assembly that captures 2.24 gigabases (Gb) in 29,454 contigs (Sup table 2c). We first assembled these contigs based on unique MP links of mapping results. The sequence number is significantly reduced from 29,454 contigs to 9,157 scaffolds, and the scaffold N50 is improved from 120.61 Kb to 491.06 Kb (Sup table 2c). To place these assemblies on super-scaffolds and determine the physical length of gaps between them, we developed a robust physical map from

2.88 million (~596.26 Gb) high quality BioNano optical molecules (Sup table 1c). The assembles and N50 size of genome map is 2.65 Gb and 3.40 Mb, respectively, consisting of 1,404 sequences (Sup table 1c). After genome curation of integrated evidence and hybrid assembly of assemblies and genome optical maps, we generated 2.62 Gb assembles, occupying 97.03% of the estimated genome size (Sup table 2c). Adopting chromatin interaction mapping (Hi-C) links, we build the sequences of the 20 pseudomolecules that anchored 91.83% of the genome content (Fig. 2 and Table 1). Referencing to the syntenic relationship between the sequences of *A.monticola* and those of its progenitors (*A. duranensis, A. ipaensis*), 96.07% of assemblies was successfully distinguished into two subgenomes (Table 1 and Sup figure 1- 2).

Here we demonstrate the current state of the art for the *de novo* assembly of a highly complex genome for the allotetraploid wild peanut (*A. monticola*), based on long reads for contig formation, short reads for consensus validation, and scaffolding by MP links, optical map and chromatin interaction mapping. These combined technologies produced reference-quality genome of tetraploid wild peanut, with chromosome-length scaffolds (Table 1 and Sup table 2b). Our assemblies represented a five-fold improvement in continuity attributing to properly assembled gaps, compared to the previously published *A. duranensis* and *A. ipaensis* assembly, and better resolved the repetitive structures longer than 10 Kb, especially the nearly identical regions of the two subgenomes (Table 2 and Sup table 2d).

Taken together, we have developed an integrated approach, including “WGS and Pacbio and BioNano optics and Hi-C”, to the sequencing and assembly of an allopolyploid *Arachis monticola* genome(Fig. 2). The final assembly comprised of 28,581contigs (N50=129.50 Kb) and 4,135 scaffolds (N50=118.65 Mb) (Sup table 2c), and can be organized into 20 chromosomes, including 1.06 Gb in the A subgenome and 1.45 Gb in the B subgenome (Table 1; Fig. 3A). Our assembly contains 97 % of the *A. monticola* genome sequence.

The *Arachis monticola* genome presented here provides, for the first time, a reference genome for future studies of this important tetraploid wild peanut, which may be the “bridge” connecting the diploid wild species and tetraploid cultivated species to study subgenomes evolution, origin and domestication among *Arachis* genus and other plants which will provide a wealth of information to enable studies of phylogeny, genome duplication, and convergent evolution [38]. The atlas data of the *A. monticola* genome will provide a valuable resource and facilitate future functional genomics and molecular-assisted breeding in this oil crop. Meanwhile, more reference information should be beneficial for studying the genetic changes during the recent polyploidization event and producing more elite peanut cultivars.

Abbreviations

BUSCO: Benchmarking Universal Single-Copy Orthologs; CEGMA: core eukaryotic gene-mapping approach; Gb: gigabase; Hi-C: High-throughput chromosome

conformation capture; Kb: kilobase; Mb: megabase; MP: meta-paired; NGM-HS: Next Generation Mapping; PacBio: Pacific Biosciences; PE: paired-end; single molecule real-time; SNP: single nucleotide polymorphism; WGS: whole genome sequencing.

Availability of supporting data

The Whole Genome Shotgun project has been deposited at DDBJ/ENA/GenBank under the accession QBTX00000000. The version described in this paper is version QBTX01000000. Raw reads, genome assembly sequences of *Arachis monticola* genome project have been deposited at the NCBI GeneBank under BioProject PRJNA430760 and BioSample Accession SAMN08378480. All supplementary figures and tables are provided in Additional Files. Supporting data including annotations and RNA-seq data are also available in the GigaDB database[39].

Additional files

Sup table 1a. Summary of Illumina data for *A. monticola*.

Sup table 1b. Statistic of PacBio sub-reads length distribution for *A. monticola*.

Sup table 1c. Summary of BioNano data collection and assembly statistics.

Sup table 1d. Summary of HiC data for error correction and chromosome construction.

Sup table 2a. Summary of assembly results from Illumina short reads.

Sup table 2b. Summary of assembly results of different tools for *A. monticola*.

Sup table 2c. Summary of assembly results of different versions for *A. monticola*.

Sup table 2d. Comparison of genome assembly between *A. monticola* and corresponding ancestors *A.duranensis* and *A.ipaensis*.

Sup table 3a. Genome completeness assessment by BUSCO.

Sup table 3b. Completeness analysis based on CEG database.

Sup table 3c. Summary of pooled transcriptome data assisted for genome annotation.

Sup table 4c.Genome completeness assessment based on sequencing reads.

Sup table 3d. Genome completeness evaluated by ESTs/unigenes.

Sup table 3e.Genome completeness assessment based on sequencing reads.

Sup table 3f. PacBio sub-reads validation for the *A. monticola* genome assembly.

Sup figure 1. Circos plot showing shared synteny between *A. monticola* and *A. duranensis.*

Sup figure 2. Circos plot showing shared synteny between *A. monticola* and *A. ipaensis.*

Competing interests

The authors declare that they have no competing interests.

Acknowledgments

This work was financially supported by grants from the National Natural Science Foundation of China (No. 31471525); Key program of NSFC-Henan United Fund（No. U1704232; key scientific and technological project in Henan Province (No.161100111000； S2012-05-G03); Innovation Scientists and Technicians Troop Construction Projects of Henan Province（No.2018JR0001）

References

1. Krapovickas, A. and W.C. Gregory, *Taxonomıía del género Arachis (Léeguminosae).* Bonplandia, 1994. **8**(1-4): p. 1-186.
2. Hilu, K.W. and H.T. Stalker, *Genetic relationships between peanut and wild species of Arachis sect. Arachis (Fabaceae): Evidence from RAPDs.* Plant Systematics & Evolution, 1995. **198**(3-4): p. 167-178.
3. Re, D., *Genetic diversity of cultivated and wild-type peanuts evaluated with M13-tailed SSR markers and sequencing.* Genetical Research, 2007. **89**(2): p. 93-106.
4. Pattee, H.E., H.T. Stalker, and F.G. Giesbrecht, *Reproductive Efficiency in Reciprocal Crosses of Arachis monticola with A. hypogaea Subspecies1.* Peanut Science, 2010. **25**(1): p. 7-12.
5. Koppolu, R., et al., *Genetic relationships among seven sections of genus Arachis studied by using SSR markers.* Bmc Plant Biology, 2010. **10**(1): p. 1-12.
6. Temsch, E.M. and J. Greilhuber, *Genome size variation in Arachis hypogaea and A. monticola re-evaluated.* Genome, 2000. **43**(3): p. 449-451.
7. Bertioli, D.J., et al., *The genome sequences of Arachis duranensis and Arachis ipaensis, the diploid ancestors of cultivated peanut.* Nature Genetics, 2016. **48**(4): p. 438.
8. Maccallum, I., et al., *ALLPATHS 2: small genomes assembled accurately and with high continuity from short paired reads.* Genome Biology, 2009. **10**(10): p. 1-10.
9. Koren, S., et al., *Canu: scalable and accurate long-read assembly via adaptive k-mer weighting and repeat separation.* Genome Research, 2017. **27**(5): p. 722.
10. Salmela, L. and E. Rivals, *LoRDEC: accurate and efficient long read error correction.* Bioinformatics, 2014. **30**(24): p. 3506-3514.
11. Chin, C., et al., *Phased diploid genome assembly with single-molecule real-time sequencing.* Nature Methods, 2016. **13**(12): p. 1050-1054.
12. *https://github.com/ruanjue/wtdbg*.
13. Simão, F.A., et al., *BUSCO: assessing genome assembly and annotation completeness with single-copy orthologs.* Bioinformatics, 2015. **31**(19): p. 3210.
14. Parra, G., K. Bradnam, and I. Korf, *CEGMA: a pipeline to accurately annotate core genes in eukaryotic genomes.* Bioinformatics, 2007. **23**(9): p. 1061-1067.
15. Walker, B.J., et al., *Pilon: An Integrated Tool for Comprehensive Microbial Variant Detection and Genome Assembly Improvement.* Plos One, 2014. **9**(11): p. e112963.
16. Chakraborty, M., et al., *Contiguous and accurate de novo assembly of metazoan genomes with modest long read coverage.* Nucleic Acids Research, 2016. **44**(19): p. 029306.
17. Lam, E.T., et al., *Genome mapping on nanochannel arrays for structural variation analysis and sequence assembly.* Nature Biotechnology, 2012. **30**(8): p. 771-776.
18. *https://bionanogenomics.com/support/software-downloads/.*
19. Li, H. and R. Durbin, *Fast and accurate short read alignment with Burrows-Wheeler transform.* Bioinformatics, 2009. **25**(14): p. 1754-60.
20. Boetzer, M., et al., *Scaffolding pre-assembled contigs using SSPACE.* Bioinformatics, 2011. **27**(4): p. 578-9.
21. Raina, S.N. and Y. Mukai, *Genomic in situ hybridization in Arachis (Fabaceae) identifies the diploid wild progenitors of cultivated (A. hypogaea) and related wild (A. monticola) peanut species.* Plant Systematics & Evolution, 1999. **214**(1-4): p. 251-262.
22. English, A.C., et al., *Mind the Gap: Upgrading Genomes with Pacific Biosciences RS*

Long-Read Sequencing Technology. PLOS ONE, 2012. **7**(11).

23. Luo, R., et al., *SOAPdenovo2: an empirically improved memory-efficient short-read de novo assembler.* Gigascience, 2012. **1**(1): p. 18.
24. Burton, J.N., et al., *Chromosome-scale scaffolding of de novo genome assemblies based on chromatin interactions.* Nature Biotechnology, 2013. **31**(12): p. 1119.
25. Bickhart, D.M., et al., *Single-molecule sequencing and chromatin conformation capture enable de novo reference assembly of the domestic goat genome.* Nature Genetics, 2017. **49**(4): p. 643.
26. Zhang, L., et al., *The Tartary Buckwheat Genome Provides Insights into Rutin Biosynthesis and Abiotic Stress Tolerance.* Mol Plant, 2017. **10**(9): p. 1224-1237.
27. Avni, R., et al., *Wild emmer genome architecture and diversity elucidate wheat evolution and domestication.* Science, 2017. **357**(6346): p. 93-97.
28. Mascher, M., et al., *A chromosome conformation capture ordered sequence of the barley genome.* Nature, 2017. **544**(7651): p. 427.
29. Martin, M., *Cutadapt removes adapter sequences from high-throughput sequencing reads.* Embnet Journal, 2011. **17**(1).
30. Servant, N., et al., *HiC-Pro: an optimized and flexible pipeline for Hi-C data processing.* Genome Biology, 2015. **16**(1): p. 259.
31. Kurtz, S., et al., *Versatile and open software for comparing large genomes.* Genome Biology, 2004. **5**(2): p. R12.
32. Haas, B.J., et al., *De novo transcript sequence reconstruction from RNA-Seq: reference generation and analysis with Trinity.* Nature Protocols, 2013. **8**(8): p. 1494.
33. Chaisson, M.J. and G. Tesler, *Mapping single molecule sequencing reads using basic local alignment with successive refinement (BLASR): application and theory.* BMC Bioinformatics, 2012. **13**(1): p. 238.
34. Grabiele, M., et al., *Genetic and geographic origin of domesticated peanut as evidenced by 5S rDNA and chloroplast DNA sequences.* Plant Systematics & Evolution, 2012. **298**(6): p. 1151-1165.
35. Stalker, H.T., et al., *Variation of isozyme patterns among Arachis species.* Tag.theoretical & Applied Genetics.theoretische Und Angewandte Genetik, 1994. **87**(6): p. 746.
36. Moretzsohn, M.C., et al., *A study of the relationships of cultivated peanut (Arachis hypogaea) and its most closely related wild species using intron sequences and microsatellite markers.* Annals of Botany, 2013. **111**(1): p. 113.
37. Bertioli, D.J., et al., *The Use of SNP Markers for Linkage Mapping in Diploid and Tetraploid Peanuts.* G3 Genesgenetics, 2014. **4**(1): p. 89-96.
38. Shifeng Cheng, et al., 10KP: A Phylodiverse Genome Sequencing Plan, *GigaScience*, 2018, doi: 10.1093/gigascience/giy013, https://doi.org/10.1093/gigascience/giy013
39. Yin, D; Ji, C; Ma, X; Li, H; Zhang, W; Li, S; liu, F; Zhao, K; Li, F; Li, K; Ning, L; He, J; Wang, Y; Zhao, F; Xie, Y; Zheng, H; Zhang, X; Zhang, Y; Zhang, J (2018): Supporting data for "Genome of an allotetraploid wild peanut Arachis monticola: a de novo assembly" GigaScience Database. http://dx.doi.org/10.5524/100453

Tables

Table1. Statistics of pseudochromosomes of *A. monticola*.

	Chr	Length (bp)	No. of gap	Gap length (bp)	Gaps ratio (%)	Anchored percent (%)
A.mon-A	A.mon-A01	118,283,061	1,961	12,923,146	10.93	4.51
	A.mon-A02	84,409,872	1,598	13,652,890	16.17	3.22
	A.mon-A03	123,011,103	2,089	18,448,429	15.00	4.69
	A.mon-A04	106,244,467	2,020	15,031,534	14.15	4.05
	A.mon-A05	123,320,146	1,950	15,552,662	12.61	4.70
	A.mon-A06	98,474,784	1,770	11,764,791	11.95	3.75
	A.mon-A07	72,108,480	1,299	7,250,302	10.05	2.75
	A.mon-A08	39,681,652	442	1,898,702	4.78	1.51
	A.mon-A09	107,717,523	1,889	11,324,084	10.51	4.11
	A.mon-A10	100,634,791	1,847	13,895,555	13.81	3.84
	Un-chr	61,870,352	422	7,811,614	12.63	2.36
A.mon-B	A.mon-B01	140,073,190	2,773	17,354,378	12.39	5.34
	A.mon-B02	124,915,013	2,271	14,941,271	11.96	4.76
	A.mon-B03	160,549,902	2,512	18,727,668	11.66	6.12
	A.mon-B04	147,957,427	2,521	16,939,677	11.45	5.64
	A.mon-B05	121,568,645	2,396	14,347,666	11.80	4.63
	A.mon-B06	154,488,041	2,644	22,222,939	14.38	5.89
	A.mon-B07	136,067,974	2,462	15,804,193	11.61	5.19
	A.mon-B08	138,850,997	2,492	17,429,178	12.55	5.29
	A.mon-B09	163,848,611	2,991	16,573,361	10.12	6.24
	A.mon-B10	147,468,805	2,693	18,369,757	12.46	5.62
	Un-chr	49,370,401	428	7,142,698	14.47	1.88
Unknown	--	103,005,886	972	16,282,706	15.81	--
	Total	2,623,921,123	46,879	325,689,201	12.41	--

Table2. Comparison of assembly results between *A. monticola* and its progenitors.

	A.mon-A	*A.mon*-B	*A. duranensis*	*A. ipaensis*
Genome size (bp)	1,035,756,231	1,485,159,006	1,068,326,401	1,257,035,815
Contig number	18,620	27,431	135,613	123,165
Max length (bp)	1,481,449	1,683,058	221,145	250,973
Min length (bp)	14,852	10,392	10,007	10,021
Contig N50 (bp)	107,702	110,501	22,900	22,562
Contig N90 (bp)	29,116	29,291	3,342	5,216
Gap number	18,005	26,847	134,110	122,617
Gap ratio (%)	12.50	12.11	11.95	7.32
GC content (%)	35.79	36.18	35.81	36.85

Note: only sequences whose length is more than 10 Kb are considered.

Figure Legends

Figure 1. Morphological characters of the *Arachis monticola*. Mature plants in field (A), flowers (B), and pods (C) are shown.

Figure 2. Work flow of assembly of alloteraploid wild peanut (*A. monticola*). We first corrected SMRT subreads by error correction module of Canu based on 36.10 x Pacbio subreads. For subreads aborted by Canu, we corrected them with LoRDEC based on ~50 fold coverage of Illumina short reads. Then we assembled these high quality data using Canu, Falcon and WTDGB, respectively, and used Pilon to polish them. To integrate advantages of different algorithm, we merged the assemblies by Quickmerge. We also curated "chimeric error" of genome assembly combing Pacbio molecules, BioNano data and HiC links, and scaffolded the contigs using SSPACE and IrysView. Further analysis of scaffolds order and orientation through HiC-pro and LACHESIS led to chromosome-length scaffolds. SMRT subreads and short reads were used for gap filling and genome polishing through Pbjelly, GapCloser and Pilon packages. The subgenomes of AA- and BB- genotypes were simply distinguished by the overall macro-synteny between genome assemblies and its corresponding ancestors.

Figure 3. Interaction frequency distribution of Hi-C links among chromosomes. (A) Genome-wide Hi-C map of *A. monticola*. (B) Genome-wide Hi-C map of *A.ipaensis* and *A.duranensis*. We scanned the genome by 500 Kb non-overlapping window as a bin and calculated valid interaction links of Hi-C data between any pair of bins. The log2 of link number was calculated. The distribution of links among chromosomes was exhibited by heatmap based on HiCplotter. The color key of heatmap ranging from light yellow to dark red indicated the frequency of Hi-C interaction links from low to high (0~10).
