# Supplementary figures and images for: Genome of an allotetraploid wild peanut Arachis monticola: a de novo assembly
Source: Gigascience. 2018 Jun 19;7(6):giy066. doi: 10.1093/gigascience/giy066 (PMC6009596; doi:10.1093/gigascience/giy066)

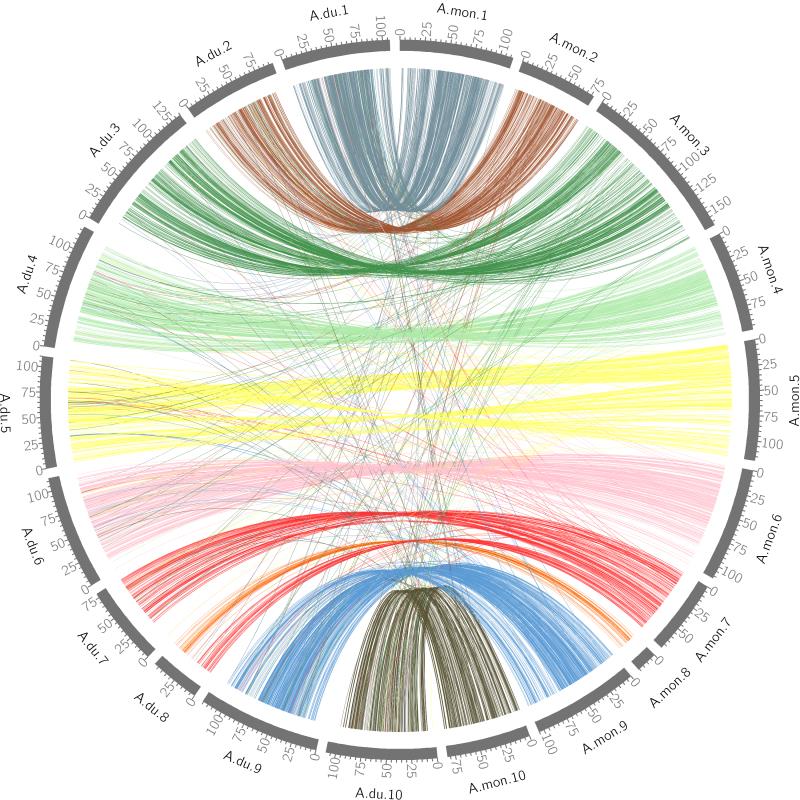

Supplement: Supplement Files [file giy066_supplement_files.zip › Sup figure 1.jpg]

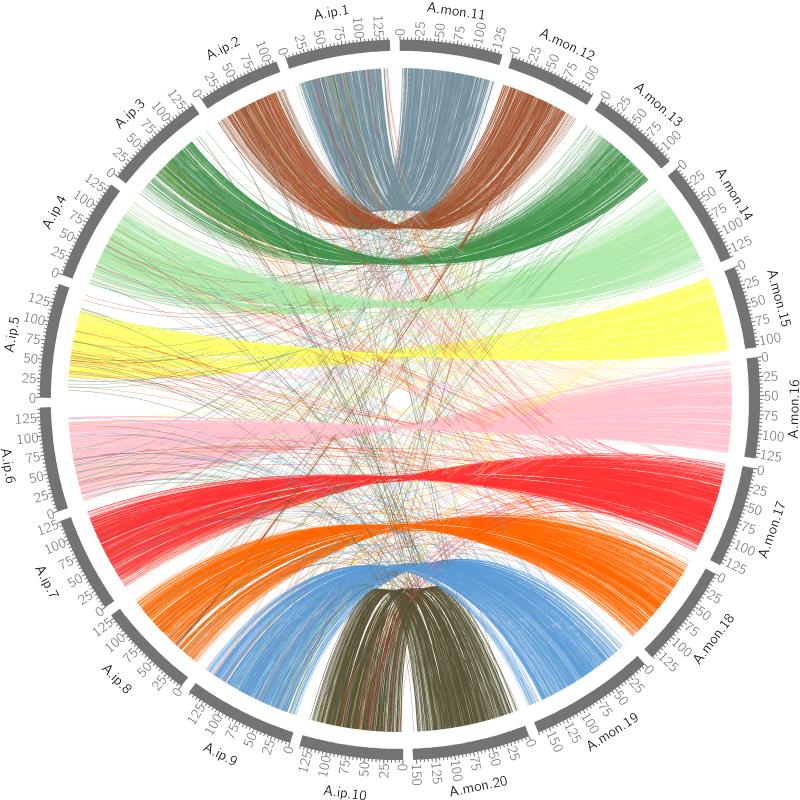

Supplement: Supplement Files [file giy066_supplement_files.zip › Sup figure 2.jpg]
