# Supplementary material for: Genome of an allotetraploid wild peanut Arachis monticola: a de novo assembly
Source: Gigascience. 2018 Jun 19;7(6):giy066. doi: 10.1093/gigascience/giy066 (PMC6009596; doi:10.1093/gigascience/giy066)
Supplement: Response_to_Reviewer_Comments_Original_Submission.pdf [file giy066_response_to_reviewer_comments_original_submission.pdf]

Author's Response To Reviewer Comments

Close

Reviewer #1: This paper reports what appears to be a high-quality genome assembly of the wild tetraploid peanut relative, Arachis monticola. The paper primarily reports the assembly methods and statistics. As far as I am able to tell, the assembly appears to have been made using state-of-the-art methods.

The main weakness (and a very large one) is that the assembly is not accessible. I assume it will be made available after embargo, but as this paper primarily describes the assembly resource, I don't feel that I can adequately assess the resource if it is not available. It appears that the accession IDs provided for GigaDB and GenBank are placeholders. I believe the standard practice is instead to provide actual identifiers provided by those databases - which may be held pending embargo release, depending on release conditions.

Response: we have provided the correct accessions in NCBI and GigaDB including project accession, biosample accession, SRA accessions of Illumina, pacbio and HiC data, and genome sequences accession in Availability of supporting data.

It would also have been nice for the authors to have provided some scientific analysis of the assembly, beyond a simple description of the resource. For example, no report is provided regarding sequence interactions (exchanges) between the subgenomes, or evidence about rearrangements in the progenitor species - or sequence similarity with the diploid progenitors.

Response: We appreciate your valuable suggestion. In this study, we released the genome of an allotetraploid wild peanut firstly for publication in Gigascience as “DATA NOTE” article, and we provided here the circus plot to demonstrate the high consistency of A and B subgenomes in tetraploid peanut with diploid ancestors (A. ipaensis and A. duranensis) (Sup Figure1-2). More detailed analysis are already under way and an interesting story will show in the following paper.

Specific comments:
Introduction
60: "has a key role on food security within the global society" -- better as "has a key role in food security globally" (on -> in and global society -> globally)

62: "The genus Arachis is unique of subterranean fruit originated" --> The genus Arachis is unique for its subterranean fruit, which originates from

82: "billion basesand" --> : billion bases and

84: occupying --> spanning

Response: we have revised the MS following the advises in line 61, 62, 99 and 101.

Availability of supporting data:
354: At http://gigadb.org, I find no record for RRID:SCR00000 or for SCR00000. This seems to be a placeholder. The correct accession will be needed.

Response: we have revised the MS following the advises in line 388 (Availability of supporting data).

355: "Raw data were deposited in the Sequence Read Archive (SRA00000) for the Arachis monticola genome." -- the accession SRA00000 seems to be a placeholder. At GenBank, I see biosample SAMN05572396 and bioproject PRJNA248910 for Arachis monticola, but from a different research group. The correct accession(s) will be needed.

Response: we have provided the correct accessions in NCBI and GigaDB including project accession, biosample accession, SRA accessions of Illumina, pacbio and HiC data, and genome sequences accession in Availability of supporting data.

Reviewer #2: The genome assembly for Arachis monticola is very important for Arachis species research and therefore peanut research will be highly benefited by this resource. I have following comments for the authors:
1. Needs proper justification that why A. monticola is so important for peanut improvement as this wild relative of cultivated peanut

Response: We agree with this comment and associated content has been added in the revised manuscript (line 62~73, 82~91).

2. Details on the genotype/accession not provided throughout MS not provided

Response: We have added the genotype/accession of the sequencing samples in MS, from line 105 to 107.

3. The information provided in the first para of Results section was again repeated in detail in rest of the sections of the Results...looked much text repetitive

Response: We have rewrote the Results and removed the redundant contents from line 90~102 of previous version to line 113~117 of revised version.

4. The major problem with this MS is that this MS appears totally descriptive in nature and no scientific advancement been provided to the available understanding/knowledge

Response: We have revised the MS as review's advises from line 62~73, 82~91 in the revised version.

5. Sequencing genome is not just enough for publications..until unless it makes some sense for the research community

Response: We follow the review's points. Although previous publication of reference genome sequences of peanut diploid ancestors (A. ipaensis and A. duranensis) provide the valuable understanding and knowledge of peanut/legume and facilitated the peanut research, all the cultivated peanut varieties are allotetraploids. The high quality reference genome of allotetraploid peanut is important for evolution, origin and domestication research of wild and cultivated peanuts, and favorable to peanut breeding, which is helpful for peanut research community. So we published the genome assembly as data note as soon as possible,

which would benefit to community and scientists. The further research including subgenomes evolution, origin and domestication also have been planned. Here, we provide the circus plot to demonstrate the high consistency of A and B subgenomes in tetraploid peanut with diploid ancestors (A. ipaensis and A. duranensis).

6. The MS in present form does not add value to much for peanut research community except providing genome assembly of A. monticola for research

Response: Please refer to the response to comment 5.

7. Authors are suggested to include some evolutionary, origin and domestication related analysis and results for improving this MS

Response: Please refer to the response to comment 5.

Close
