# Supplementary material for: Genome of an allotetraploid wild peanut Arachis monticola: a de novo assembly
Source: Gigascience. 2018 Jun 19;7(6):giy066. doi: 10.1093/gigascience/giy066 (PMC6009596; doi:10.1093/gigascience/giy066)
Supplement: Response_to_Reviewer_Comments_Revision_1.pdf [file giy066_response_to_reviewer_comments_revision_1.pdf]

Author's Response To Reviewer Comments

Close

Reviewer reports:

Reviewer #1: The remaining major problem with this manuscript is the data availability.

For the BioProject, it appears that only the raw SRA reads are available:
https://www.ncbi.nlm.nih.gov/bioproject/PRJNA430760

I find no record "SCR_004002" at gigadb.org

If this paper describes a genome assembly, then the genome assembly needs to be available - not just the raw reads. The assembly - as described in the paper, with named pseudomolecules plus remaining scaffolds - needs to be available at the time of review? In my opinion, the paper will only be acceptable once the assembly that is described in the paper is available - ideally, in an INSDC-compliant repository (DDBJ/ENA/GenBank). On this basis, I recommend "Major Revision."

Response: We appreciate your valuable suggestion. Thanks a lot for your kindly help and earnest work to improve our manuscript. Due to the importance and complexity of the project, this work had been done for many years.
Now this Whole Genome Shotgun project has been deposited at DDBJ/ENA/GenBank under the accession QBTX00000000. The version described in this paper is version QBTX01000000. The genome assembly is also available in "GigaDB".

Other, more minor issues:
Abstract
45 "assembly of A. momticola genome" --> assembly of the A. momticola genome
Response: Thanks a lot. We have revised it following the advises in line 45.

46 "~2.62 Gb representing 97.04%" -- Because of uncertainty in the estimate of the actual genome size (~2.7 Gb), four significant digits (97.04%) in the estimate of the proportion of the genome sequenced is almost certainly not warranted. For example, if the true genome size is 2.75 Gb, then 2.62/2.75 = 0.95 captured.
Response: The genome of wild peanut A. monticola genome is about 2.7 Gb according to the references below.
Temsch, E.M. and J. Greilhuber, Genome size variation in Arachis hypogaea and A. monticola re-evaluated. Genome, 2000. 43(3): p. 449-451.
Bertioli, D.J., et al., The genome sequences of Arachis duranensis and Arachis ipaensis, the diploid ancestors of cultivated peanut. Nature Genetics, 2016. 48(4): p. 438.

Introduction
66 wildspecies --> wild species
Response: Thanks a lot. We have revised it following the advises in line 66.

67-68 "A. monticola was identical to the accession of A. hypogaea with high genetic identity" -- this doesn't make sense. There would be particular, distinct accessions of A.

monticola - and these should be distinct from accessions of A. hypogaea.
Response: Thanks a lot. We have revised it following the advises in line 67.

69-71 "Arachis monticola is considered a distinct species from A. hypogaea based mainly on its fruit structure, which has an isthmus separating each seed." -- This sentence comes verbatim from Moretzsohn et al. (2013; https://doi.org/10.1093/aob/mcs237). Several other sentences in the introduction also appear to come from this paper (with minor modification). This section doesn't cite that paper (though the paper is cited in the Discussion).
Response: We have added the reference in this section.

100-101 "Finally, we generated 2.62 Gb assembles" --> "Finally, we generated a 2.62 Gb assembly
Response: Thanks a lot. We have revised it following the advises in line 100-101.

101 "spanning 97.04 % of the estimated genome size for A. monticola" -- see comments above regarding significant digits with respect to the estimated genome size.
Response: The genome of wild peanut A. monticola genome is about 2.7 Gb according to the references below.
Temsch, E.M. and J. Greilhuber, Genome size variation in Arachis hypogaea and A. monticola re-evaluated. Genome, 2000. 43(3): p. 449-451.
Bertioli, D.J., et al., The genome sequences of Arachis duranensis and Arachis ipaensis, the diploid ancestors of cultivated peanut. Nature Genetics, 2016. 48(4): p. 438.

Results
105 "We selected the seeds of PI263393 lines for genome sequencing" --> Line PI 263393 was selected for genome sequencing (PI 263393 is a single line or accession). PI lines are usually indicated with a space between PI and the number.
Response: Thanks a lot. We have revised it following the advises in line 105.

113 "Take advantage of integrated technologies" --> Taking advantage of integrated technologies
Response: Thanks a lot. We have revised it following the advises in line 113.

Methods

177 "To decrease the chimeric in initial" --> To decrease chimeras in the initial
Response: Thanks a lot. We have revised it following the advises in line 177.

266 "Benefited from the published genomes of A. duranensis and A. ipaensis" --> Benefiting from the published genomes of A. duranensis and A. ipaensis
Response: Thanks a lot. We have revised it following the advises in line 266.

356 "We demonstrated current state of the strategy" -- the more common colloquial term is "current state of the art"
Response: The word “art” is fascinating. We have revised it in line 356.

413 "Circus plot" --> Circos plot
415 "Circus plot" --> Circos plot
Response: Thanks a lot. We have revised it following the advises in line 415 and 417.

Close
