# Supplementary material for: Genome of an allotetraploid wild peanut Arachis monticola: a de novo assembly
Source: Gigascience. 2018 Jun 19;7(6):giy066. doi: 10.1093/gigascience/giy066 (PMC6009596; doi:10.1093/gigascience/giy066)
Supplement: Response_to_Reviewer_Comments_Revision_2.pdf [file giy066_response_to_reviewer_comments_revision_2.pdf]

Author's Response To Reviewer Comments

Close

Reviewer reports:
Reviewer #1: Thanks to the authors for the last round of revisions. The remaining significant issue has been addressed - that is, submission of the assembly to GenBank.

There is still a very minor point at lines 46-47 - something that I think should be at the author's discretion, but I mention for completeness. My point was not that the estimate of 97.04% was necessarily incorrect, but that the use of four significant digits is unwarranted (it is impossible to know whether 97.04% is correct given the data), because the size estimate for the genome has low precision: "about 2.7 Gb". I just point it out as a suggestion to clean up something that looks unprofessional. See the discussion of significant digits here for example: http://ec.europa.eu/eurostat/statistics-explained/index.php/Tutorial:Rounding_of_numbers

Response： We really appreciate your kind help for improving our manuscript. We have deleted the setence" representing 97.04% of the estimated genome size" following the advises in line 46-47.

Close
