# Supplementary material for: Genome of an allotetraploid wild peanut Arachis monticola: a de novo assembly
Source: Gigascience. 2018 Jun 19;7(6):giy066. doi: 10.1093/gigascience/giy066 (PMC6009596; doi:10.1093/gigascience/giy066)
Supplement: Reviewer_1_Report_(Original_Submission) -- Steven Cannon — 2/13/2018 Reviewed [file giy066_reviewer_1_report_(original_submission).pdf]

Reviewer Report

Title: **Genome of an allotetraploid wild peanut Arachis monticola: a de novo assembly**

Version: **Original Submission** **Date:** 2/13/2018

Reviewer name: **Steven Cannon**

Reviewer Comments to Author:

This paper reports what appears to be a high-quality genome assembly of the wild tetraploid peanut relative, Arachis monticola. The paper primarily reports the assembly methods and statistics. As far as I am able to tell, the assembly appears to have been made using state-of-the-art methods. The main weakness (and a very large one) is that the assembly is not accessible. I assume it will be made available after embargo, but as this paper primarily describes the assembly resource, I don't feel that I can adequately assess the resource if it is not available. It appears that the accession IDs provided for GigaDB and GenBank are placeholders. I believe the standard practice is instead to provide actual identifiers provided by those databases - which may be held pending embargo release, depending on release conditions.It would also have been nice for the authors to have provided some scientific analysis of the assembly, beyond a simple description of the resource. For example, no report is provided regarding sequence interactions (exchanges) between the subgenomes, or evidence about rearrangements in the progenitor species - or sequence similarity with the diploid progenitors.Specific comments:Introduction60: "has a key role on food security within the global society" -- better as "has a key role in food security globally" (on -> in and global society -> globally)62: "The genus Arachis is unique of subterranean fruit originated" --> The genus Arachis is unique for its subterranean fruit, which originates from82: "billion basesand" --> : billion bases and84: occupying --> spanningAvailability of supporting data:354: At http://gigadb.org, I find no record for RRID:SCR00000 or for SCR00000. This seems to be a placeholder. The correct accession will be needed.355: "Raw data were deposited in the Sequence Read Archive (SRA00000) for the Arachis monticola genome." -- the accession SRA00000 seems to be a placeholder. At GenBank, I see biosample SAMN05572396 and bioproject PRJNA248910 for Arachis monticola, but from a different research group. The correct accession(s) will be needed.

Level of Interest

Please indicate how interesting you found the manuscript: An article of importance in its field

Quality of Written English

Please indicate the quality of language in the manuscript: Needs some language corrections before being published

Declaration of Competing Interests

Please complete a declaration of competing interests, considering the following questions:

- Have you in the past five years received reimbursements, fees, funding, or salary from an organisation that may in any way gain or lose financially from the publication of this manuscript, either now or in the future?

- Do you hold any stocks or shares in an organisation that may in any way gain or lose financially from the publication of this manuscript, either now or in the future?
- Do you hold or are you currently applying for any patents relating to the content of the manuscript?
- Have you received reimbursements, fees, funding, or salary from an organization that holds or has applied for patents relating to the content of the manuscript?
- Do you have any other financial competing interests?
- Do you have any non-financial competing interests in relation to this paper?

If you can answer no to all of the above, write 'I declare that I have no competing interests' below. If your reply is yes to any, please give details below.

I declare that I have no competing interests

I agree to the open peer review policy of the journal. I understand that my name will be included on my report to the authors and, if the manuscript is accepted for publication, my named report including any attachments I upload will be posted on the website along with the authors' responses. I agree for my report to be made available under an Open Access Creative Commons CC-BY license (http://creativecommons.org/licenses/by/4.0/). I understand that any comments which I do not wish to be included in my named report can be included as confidential comments to the editors, which will not be published.

I agree to the open peer review policy of the journal

To further support our reviewers, we have joined with Publons, where you can gain additional credit to further highlight your hard work (see: https://publons.com/journal/530/gigascience). On publication of this paper, your review will be automatically added to Publons, you can then choose whether or not to claim your Publons credit. I understand this statement. Yes
