# Supplementary material for: Genome of an allotetraploid wild peanut Arachis monticola: a de novo assembly
Source: Gigascience. 2018 Jun 19;7(6):giy066. doi: 10.1093/gigascience/giy066 (PMC6009596; doi:10.1093/gigascience/giy066)
Supplement: Reviewer_1_Report_(Revision_1) -- Steven Cannon — 4/4/2018 Reviewed [file giy066_reviewer_1_report_(revision_1).pdf]

Reviewer Report

Title: **Genome of an allotetraploid wild peanut Arachis monticola: a de novo assembly**

Version: **Revision 1** **Date:** 4/4/2018

Reviewer name: **Steven Cannon**

Reviewer Comments to Author:

The remaining major problem with this manuscript is the data availability.For the BioProject, it appears that only the raw SRA reads are available:https://www.ncbi.nlm.nih.gov/bioproject/PRJNA430760I find no record "SCR_004002" at gigadb.orgIf this paper describes a genome assembly, then the genome assembly needs to be available - not just the raw reads. The assembly - as described in the paper, with named pseudomolecules plus remaining scaffolds - needs to be available at the time of review? In my opinion, the paper will only be acceptable once the assembly that is described in the paper is available - ideally, in an INSDC-compliant repository (DDBJ/ENA/GenBank). On this basis, I recommend "Major Revision."Other, more minor issues:Abstract45 "assembly of A. momticola genome" --> assembly of the A. momticola genome46 "~2.62 Gb representing 97.04%" -- Because of uncertainty in the estimate of the actual genome size (~2.7 Gb), four significant digits (97.04%) in the estimate of the proportion of the genome sequenced is almost certainly not warranted. For example, if the true genome size is 2.75 Gb, then 2.62/2.75 = 0.95 captured.Introduction66 wildspecies --> wild species67-68 "A. monticola was identical to the accession of A. hypogaea with high genetic identity" -- this doesn't make sense. There would be particular, distinct accessions of A. monticola - and these should be distinct from accessions of A. hypogaea.69-71 "Arachis monticola is considered a distinct species from A. hypogaea based mainly on its fruit structure, which has an isthmus separating each seed." -- This sentence comes verbatim from Moretzsohn et al. (2013; https://doi.org/10.1093/aob/mcs237). Several other sentences in the introduction also appear to come from this paper (with minor modification). This section doesn't cite that paper (though the paper is cited in the Discussion).100-101 "Finally, we generated 2.62 Gb assembles" --> "Finally, we generated a 2.62 Gb assembly101 "spanning 97.04 % of the estimated genome size for A. monticola" -- see comments above regarding significant digits with respect to the estimated genome size.Results105 "We selected the seeds of PI263393 lines for genome sequencing" --> Line PI 263393 was selected for genome sequencing (PI 263393 is a single line or accession). PI lines are usually indicated with a space between PI and the number.113 "Take advantage of integrated technologies" --> Taking advantage of integrated technologiesMethods177 "To decrease the chimeric in initial" --> To decrease chimeras in the initial266 "Benefited from the published genomes of A. duranensis and A. ipaensis" --> Benefiting from the published genomes of A. duranensis and A. ipaensis 356 "We demonstrated current state of the strategy" -- the more common colloquial term is "current state of the art"413 "Circus plot" --> Circos plot415 "Circus plot" --> Circos plot

Level of Interest

Please indicate how interesting you found the manuscript: An article whose findings are important to those with closely related research interests

Quality of Written English

Please indicate the quality of language in the manuscript: Acceptable

Declaration of Competing Interests

Please complete a declaration of competing interests, considering the following questions:

- Have you in the past five years received reimbursements, fees, funding, or salary from an organisation that may in any way gain or lose financially from the publication of this manuscript, either now or in the future?
- Do you hold any stocks or shares in an organisation that may in any way gain or lose financially from the publication of this manuscript, either now or in the future?
- Do you hold or are you currently applying for any patents relating to the content of the manuscript?
- Have you received reimbursements, fees, funding, or salary from an organization that holds or has applied for patents relating to the content of the manuscript?
- Do you have any other financial competing interests?
- Do you have any non-financial competing interests in relation to this paper?

If you can answer no to all of the above, write 'I declare that I have no competing interests' below. If your reply is yes to any, please give details below.

I declare that I have no competing interests

I agree to the open peer review policy of the journal. I understand that my name will be included on my report to the authors and, if the manuscript is accepted for publication, my named report including any attachments I upload will be posted on the website along with the authors' responses. I agree for my report to be made available under an Open Access Creative Commons CC-BY license (http://creativecommons.org/licenses/by/4.0/). I understand that any comments which I do not wish to be included in my named report can be included as confidential comments to the editors, which will not be published.

I agree to the open peer review policy of the journal

To further support our reviewers, we have joined with Publons, where you can gain additional credit to further highlight your hard work (see: https://publons.com/journal/530/gigascience). On publication of this paper, your review will be automatically added to Publons, you can then choose whether or not to claim your Publons credit. I understand this statement. Yes
