# Supplementary material for: Genome of an allotetraploid wild peanut Arachis monticola: a de novo assembly
Source: Gigascience. 2018 Jun 19;7(6):giy066. doi: 10.1093/gigascience/giy066 (PMC6009596; doi:10.1093/gigascience/giy066)
Supplement: Reviewer_2_Report_(Original_Submission) -- Manish Kumar Pandey, Ph.D. — 2/23/2018 Reviewed [file giy066_reviewer_2_report_(original_submission).pdf]

Reviewer Report

Title: Genome of an allotetraploid wild peanut Arachis monticola: a de novo assembly

Version: Original Submission **Date:** 2/23/2018

Reviewer name: Manish Kumar Pandey, Ph.D.

Reviewer Comments to Author:

The genome assembly for Arachis monticola is very important for Arachis species research and therefore peanut research will be highly benefitted by this resource. I have following comments for the authors: 1. Needs proper justification that why A. monticola is so important for peanut improvement as this wild relative of cultivated peanut2. Details on the genotype/accession not provided throughout MS not provided3. The information provided in the first para of Results section was again repeated in detail in rest of the sections of the Results...looked much text repetitive4. The major problem with this MS is that this MS appears totally descriptive in nature and no scientific advancement been provided to the available understanding/knowledge5. Sequencing genome is not just enough for publications..until unless it makes some sense for the research community6. The MS in present form does not add value to much for peanut research community except providing genome assembly of A. monticola for research7. Authors are suggested to include some evolutionary, origin and domestication related analysis and results for improving this MS

Level of Interest

Please indicate how interesting you found the manuscript: An article of importance in its field

Quality of Written English

Please indicate the quality of language in the manuscript: Needs some language corrections before being published

Declaration of Competing Interests

Please complete a declaration of competing interests, considering the following questions:

- Have you in the past five years received reimbursements, fees, funding, or salary from an organisation that may in any way gain or lose financially from the publication of this manuscript, either now or in the future?
- Do you hold any stocks or shares in an organisation that may in any way gain or lose financially from the publication of this manuscript, either now or in the future?
- Do you hold or are you currently applying for any patents relating to the content of the manuscript?
- Have you received reimbursements, fees, funding, or salary from an organization that holds or has applied for patents relating to the content of the manuscript?
- Do you have any other financial competing interests?

- Do you have any non-financial competing interests in relation to this paper?

If you can answer no to all of the above, write 'I declare that I have no competing interests' below. If your reply is yes to any, please give details below.

I declare that I have no competing interests

I agree to the open peer review policy of the journal. I understand that my name will be included on my report to the authors and, if the manuscript is accepted for publication, my named report including any attachments I upload will be posted on the website along with the authors' responses. I agree for my report to be made available under an Open Access Creative Commons CC-BY license (http://creativecommons.org/licenses/by/4.0/). I understand that any comments which I do not wish to be included in my named report can be included as confidential comments to the editors, which will not be published.

I agree to the open peer review policy of the journal

To further support our reviewers, we have joined with Publons, where you can gain additional credit to further highlight your hard work (see: https://publons.com/journal/530/gigascience). On publication of this paper, your review will be automatically added to Publons, you can then choose whether or not to claim your Publons credit. I understand this statement. Yes
